# Supplementary material for: Pulmonary Epithelial Cell-Derived Cytokine TGF-β1 Is a Critical Cofactor for Enhanced Innate Lymphoid Cell Function
Source: Immunity. 2015 Nov 17;43(5):945–58. doi: 10.1016/j.immuni.2015.10.012 (PMC4658339; doi:10.1016/j.immuni.2015.10.012)
Supplement: Document S1. Figures S1–S6 [file mmc1.pdf]

Immunity

Supplemental Information

Pulmonary Epithelial Cell-Derived Cytokine TGF-β1 Is a Critical Cofactor for Enhanced Innate Lymphoid Cell Function

Laura Denney, Adam J. Byrne, Thomas J. Shea, James S. Buckley, James E. Pease, Gaelle M.F. Herledan, Simone A. Walker, Lisa G. Gregory, and Clare M. Lloyd

Figure S1, related to Figure 1. Schematic of the generation of epithelial TGF-β knockout mice and the subsequent expression of Cre and TGF-β1 in the lung. (A) Double transgenic mice *CCSP*-$_{\text{rtTA/tetO}}$-Cre are crossed with mice expressing homozygous *Tgfb1* genes with LoxP sites (Floxed) and treated with doxycycline (DOX) at 6weeks of age. Upon DOX administration the Tet-on system is activated and cells expressing club cell secretory protein (CCSP) transcribe Cre recombinase which binds LoxP sites and excises exon 6 of the *Tgfb1* gene leading to the transcription of truncated and non-functional TGF-β. Littermate control mice lack one transgenic allele and are without a functional Tet-on system so therefore after DOX treatment continue to express TGF-β. (B) Immunohistochemistry images showing Cre expression 48hr post DOX (or mock) treatment of transgenic mice and littermate controls. (C) Immunohistochemistry images showing TGF-β expression 72hr post Dox treatment of transgenic mice and littermate controls. (D) mRNA levels of *Tgfb1* in sorted club cells ($CCSP^{+}CD45^{neg}$) and $CCSP^{neg}CD45^{+}$ cells in control and *Ccsp*-cre*Tgfb1*$^{-/-}$ mice. n=3 groups of >5 mice per group. Mann–Whitney *P<0.05. (E-H) Cell counts in the (E) blood, (F) spleen, (G) bone marrow and (H) mediastenial lymph nodes in *Ccsp*-cre*Tgfb1*$^{-/-}$ mice (DOX) and *Tgfb1*$^{+/+}$ (mock) treated mice 7, 21 and 42 days after treatment. Box and whisker plots depict the median and IQR and minimum and maximum values. Bar charts are expressed as mean +/- SEM. n=4-5 mice per group (Mann–Whitney).

Figure S2, related to Figure 1. Doxycycline administration alone does not result in an altered allergic lung phenotype (A) *Tgfb1*, *Tgfb2*, and *Tgfb3* mRNA levels in whole lung tissue of *Ccsp*-cre*Tgfb1*$^{-/-}$ mice (DOX) and *Tgfb1*$^{+/+}$ (mock) treated mice after HDM administration. Representative data is shown from 2 independent experiments with a total of n=8-12 mice per group (Mann–Whitney). (B-C) Airway hyperreactivity after intranasal house dust mite (HDM) or PBS administration as measured by (B) resistance and (C) compliance to an ascending methacholine concentration challenge in littermate control mice lacking either *CCSP*-$_{\mathrm{rtTA}}$ or $_{\mathrm{tetO}}$-Cre unable (upon DOX administration) to ablate TGF–β expression in their epithelium (baseline; BL). (D-F) Cell counts in the (D) BAL and (E) lung tissue after Dox treatment and HDM exposure and (F) Numbers of macrophages (MAC), eosinophils (EOS) and neutrophils (NEU) in the airways of littermate control mice lacking either *CCSP*-$_{\mathrm{rtTA}}$ or $_{\mathrm{tetO-}}$Cre. (G) Airway hyperreactivity measured by airway compliance to ascending methacholine concentration in *Ccsp*-cre*Tgfb1*$^{-/-}$ mice (DOX) and *Tgfb1*$^{+/+}$ (mock) treated mice (baseline; BL) (H) Histological scoring of lung inflammation after HDM (or PBS) treatment. (I) Eosinophils, macrophages and neutrophils in the lung tissue. Box and whisker plots depict the median and IQR and minimum and maximum values. Line graphs and bar graphs are expressed as mean +/- SEM. n=4-6 mice per group (Mann–Whitney).

Figure S3, related to Figures 2 and 3. Phenotype of *Ccsp*-cre*Tgfb1*$^{-/-}$ mice treated with HDM. (A-C) Levels of (A) KC, (B) MIP-2 and (C) MCP-1 in the lung tissue. (D) IgA levels in the BAL. (E) Flow cytometry plots show Th2 ($CD3^+CD4^+IL\text{-}13^+$) cell gating strategy example shown is HDM treated mouse lung. (F-G) Frequencies of pulmonary T cells (F) IL-4$^+$ T cells in the lung and (G) Th17 cells and (H) Th1 cells in the BAL. (I-J) Levels of (I) IL-17A and (J) IFN-γ levels in the lung. Representative data is shown from 2 independent experiments, with a total of n=8-12 mice per group, Mann–Whitney *P<0.05. Box and whisker plots depict the median and IQR and minimum and maximum values.

Figure S4, related to Figure 3. Innate lymphoid cell identification and gating. (A) Flow cytometry gating strategy for IL-13$^+$ Lin^{neg}ICOS$^+$CD45$^+$ ILCs where lineage cocktail contains CD3, CD45R, CD11b, TER-119, Ly-6G (GR1). (B) Flow cytometry gating strategy for IL-13$^+$, IL-4$^+$ and IL-5$^+$ Lin^{neg}ICOS$^+$CD45$^+$ ILCs. (C) Flow cytometry plots depicting Lin^{neg} and Lin$^+$ populations with additional linage markers (CD5, CD4, NK1.1, CD8, CD11c, β-TCR, $\gamma\delta$-TCR. (D) Plots show expression of ST2, CD127 and Thy1 on IL-13$^+$ Lin^{neg}ICOS$^+$CD45$^+$ ILC population.

Figure S5, related to Figures 3 and 4. Innate cells in the lung (A) Flow cytometry plots depicting IL-5^{+} and IL-13^{+} ILCs from PBS treated control mice and HDM treated *Ccsp*-cre*Tgfb1*$^{-/-}$ mice and littermate controls. (B) IL-4^{+} ILC numbers in the lung. (C) IL-5^{+} ILC numbers in the lung. (D-E) GATA-3^{+} ILCs in the (D) lung and (E) BAL. (F) IL-33 levels in the lung. (G) NK cell (NKp46^{+} CD3^{neg}) numbers in lung tissue of *Ccsp*-cre*Tgfb1*$^{-/-}$ mice (DOX) and *Tgfb1*$^{+/+}$ (mock) treated mice. (H) Frequency of ILC3 defined as IL-17A^{+} lineage^{neg}CD45^{+}ICOS^{+} in lung. (I) Histogram showing T1/ST2 expression on club cells (CCSP^{+}CD45^{neg}) recovered from the lung of naïve mice. (J) Levels of bioactive TGF-β in the BAL of control and *Ccsp*-cre*Tgfb1*$^{-/-}$ mice. Representative data is shown from 2 independent experiments, with a total of n=8-12 mice per group Mann–Whitney *P<0.05 and **P<0.01 Box and whisker plots depict the median and IQR and minimum and maximum values.

Figure S6, related to Figures 4 and 7. Phenotype of *Ccsp*-cre*Tgfb1*$^{-/-}$ mice mice treated with rIL-33. (A) Eosinophils (EOS), macrophages (MAC) and neutrophils (NEU) in the lung tissue. (B) Levels of IL-5 and (C) eotaxin-2 in the lung. (D) Total cell counts and (E) eosinophil numbers in the BAL. (F) Th2 cells and (G) ILC2 numbers in the BAL. (H) IL-13 levels in the BAL. (I) Accumulated distance and (J) track velocity of ILCs from control (*Tgfb1*$^{+/+}$) and *Ccsp*-cre*Tgfb1*$^{-/-}$ mice exposed to gradients of rTGF-β1 (5μg/ml) assessed using TAXIScan methodology. Representative data is shown from 2 independent experiments, with a total of n=8-12 mice per group. Box and whisker plots depict the median and IQR and minimum and maximum values. Bar graphs are expressed as mean +/- SEM.

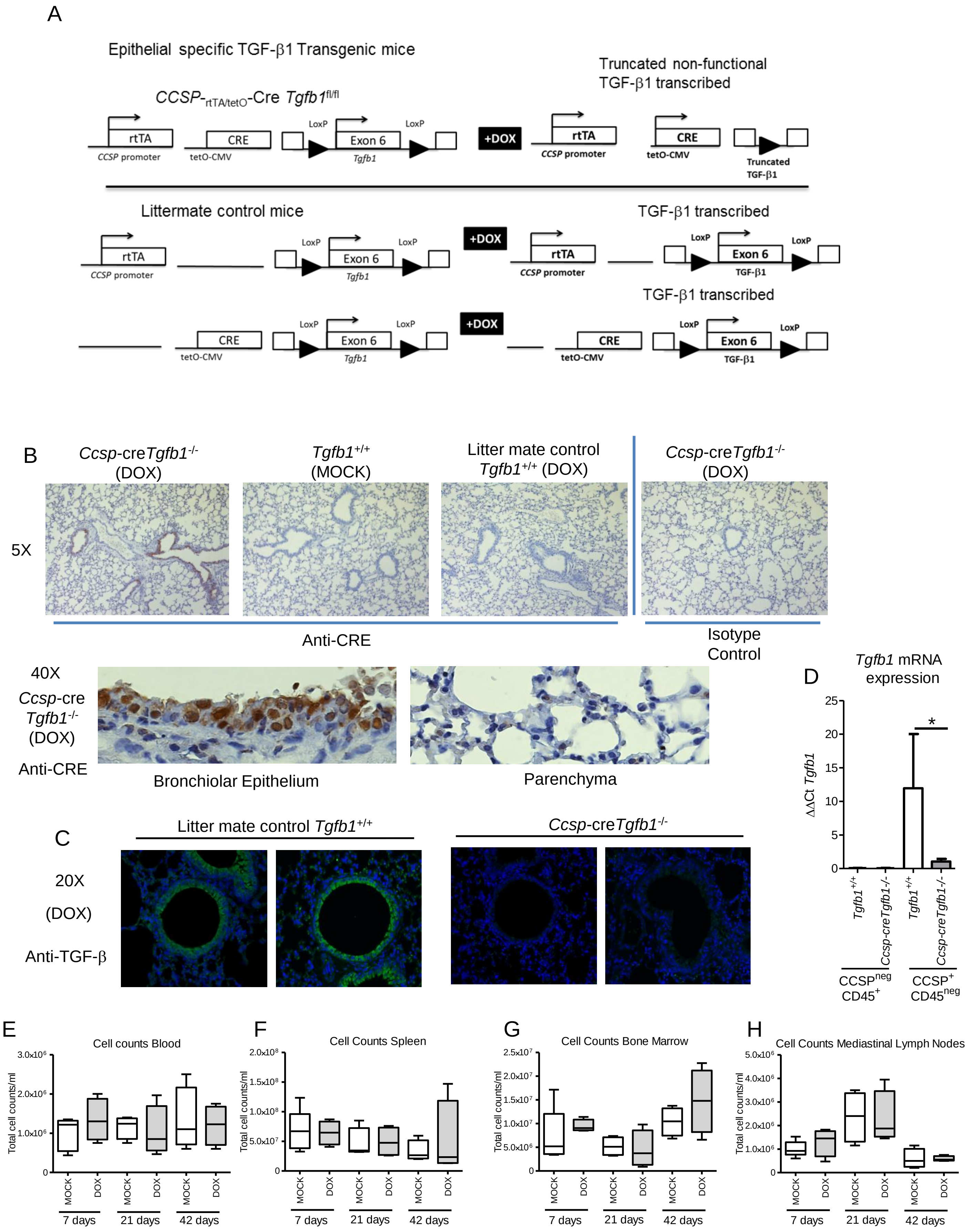
A
Epithelial specific TGF-β1 Transgenic mice
CCSP-rtTA/tetO-Cre Tgfb1fl/fl
Truncated non-functional TGF-β1 transcribed
rtTA
CCSP promoter
CRE
tetO-CMV
LoxP
Exon 6
Tgfb1
+DOX
Truncated TGF-β1
Littermate control mice
TGF-β1 transcribed
TGF-β1
B
Ccsp-creTgfb1-/- (DOX)
Tgfb1+/+ (MOCK)
Litter mate control Tgfb1+/+ (DOX)
5X
Anti-CRE
Isotype Control
40X
Bronchiolar Epithelium
Parenchyma
C
Litter mate control Tgfb1+/+
Ccsp-creTgfb1-/-
20X
(DOX)
Anti-TGF-β
D
Tgfb1 mRNA expression
ΔΔCt Tgfb1
Tgfb1+/+
Ccsp-creTgfb1-/-
CCSPneg CD45+
CCSP+ CD45neg
E
Cell counts Blood
F
Cell Counts Spleen
G
Cell Counts Bone Marrow
H
Cell Counts Mediastinal Lymph Nodes
Total cell counts/ml
MOCK
DOX
7 days
21 days
42 days

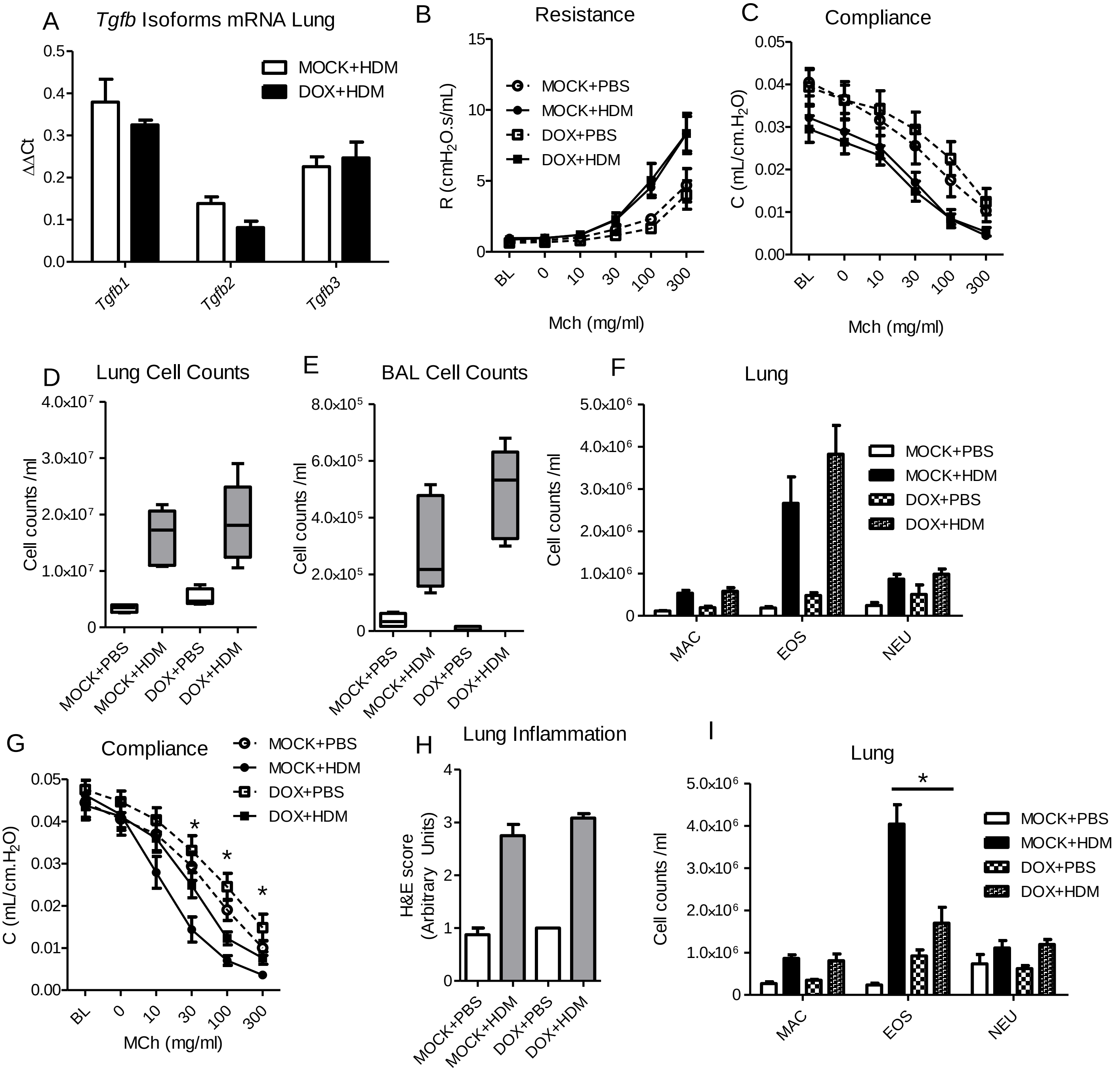
A
Tgfb Isoforms mRNA Lung
MOCK+HDM
DOX+HDM
ΔΔCt
Tgfb1
Tgfb2
Tgfb3
B
Resistance
MOCK+PBS
MOCK+HDM
DOX+PBS
DOX+HDM
R (cmH2O.s/mL)
Mch (mg/ml)
C
Compliance
C (mL/cm.H2O)
Mch (mg/ml)
D
Lung Cell Counts
Cell counts /ml
E
BAL Cell Counts
Cell counts /ml
F
Lung
Cell counts /ml
MAC
EOS
NEU
G
Compliance
C (mL/cm.H2O)
MCh (mg/ml)
H
Lung Inflammation
H&E score (Arbitrary Units)
I
Lung
Cell counts /ml

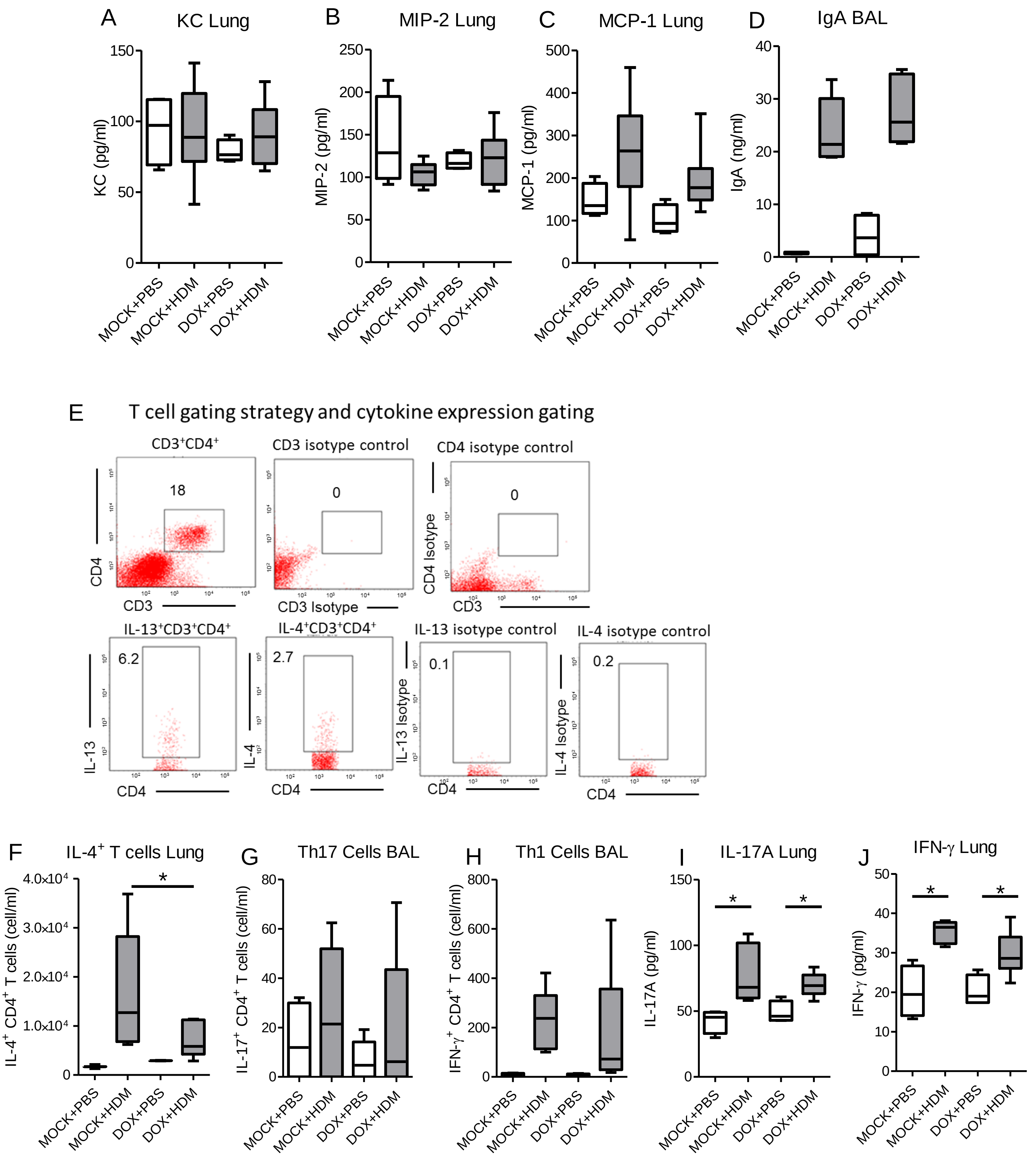

A
KC Lung
KC (pg/ml)
B
MIP-2 Lung
MIP-2 (pg/ml)
C
MCP-1 Lung
MCP-1 (pg/ml)
D
IgA BAL
IgA (ng/ml)
MOCK+PBS
MOCK+HDM
DOX+PBS
DOX+HDM
E
T cell gating strategy and cytokine expression gating
CD3+CD4+
CD3 isotype control
CD4 isotype control
CD4
CD3
CD3 Isotype
CD4 Isotype
18
0
0
IL-13+CD3+CD4+
IL-4+CD3+CD4+
IL-13 isotype control
IL-4 isotype control
6.2
2.7
0.1
0.2
IL-13
IL-4
IL-13 Isotype
IL-4 Isotype
F
IL-4+ T cells Lung
IL-4+ CD4+ T cells (cell/ml)
G
Th17 Cells BAL
IL-17+ CD4+ T cells (cell/ml)
H
Th1 Cells BAL
IFN-γ+ CD4+ T cells (cell/ml)
I
IL-17A Lung
IL-17A (pg/ml)
J
IFN-γ Lung
IFN-γ (pg/ml)
*

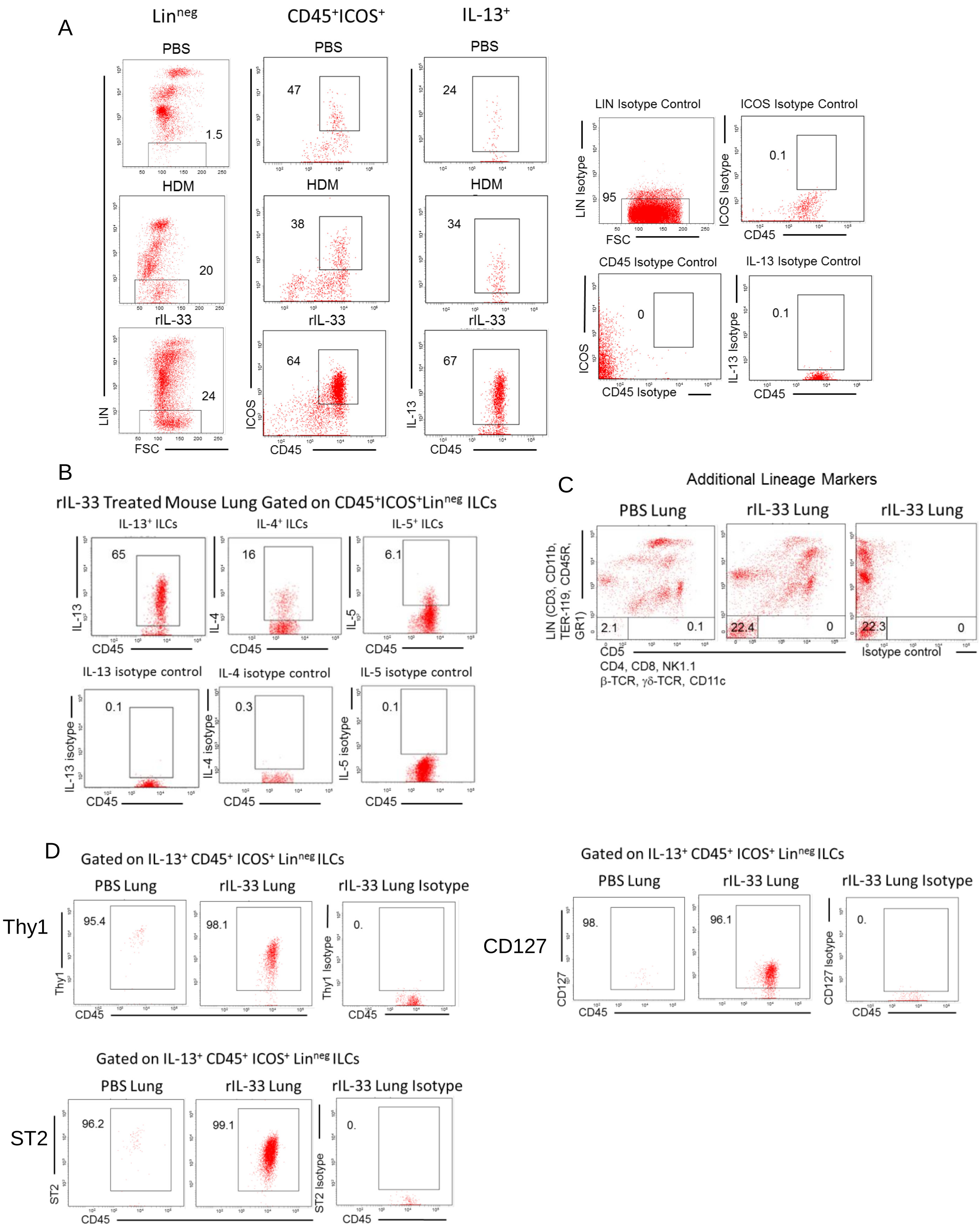
A
Lin^neg
CD45+ICOS+
IL-13+
PBS
HDM
rIL-33
1.5
20
24
47
38
64
24
34
67
LIN
FSC
ICOS
CD45
IL-13
LIN Isotype Control
ICOS Isotype Control
95
0.1
LIN Isotype
ICOS Isotype
CD45 Isotype Control
IL-13 Isotype Control
0
0.1
CD45 Isotype
IL-13 Isotype
B
rIL-33 Treated Mouse Lung Gated on CD45+ICOS+Lin^neg ILCs
IL-13+ ILCs
IL-4+ ILCs
IL-5+ ILCs
65
16
6.1
IL-4
IL-5
IL-13 isotype control
IL-4 isotype control
IL-5 isotype control
0.1
0.3
0.1
IL-13 isotype
IL-4 isotype
IL-5 isotype
C
Additional Lineage Markers
PBS Lung
rIL-33 Lung
rIL-33 Lung
LIN (CD3, CD11b, TER-119, CD45R, GR1)
2.1
0.1
22.4
0
22.3
0
CD5
CD4, CD8, NK1.1
β-TCR, γδ-TCR, CD11c
Isotype control
D
Gated on IL-13+ CD45+ ICOS+ Lin^neg ILCs
PBS Lung
rIL-33 Lung
rIL-33 Lung Isotype
Thy1
95.4
98.1
0.
Thy1 Isotype
CD127
98.
96.1
0.
CD127 Isotype
ST2
96.2
99.1
0.
ST2 Isotype

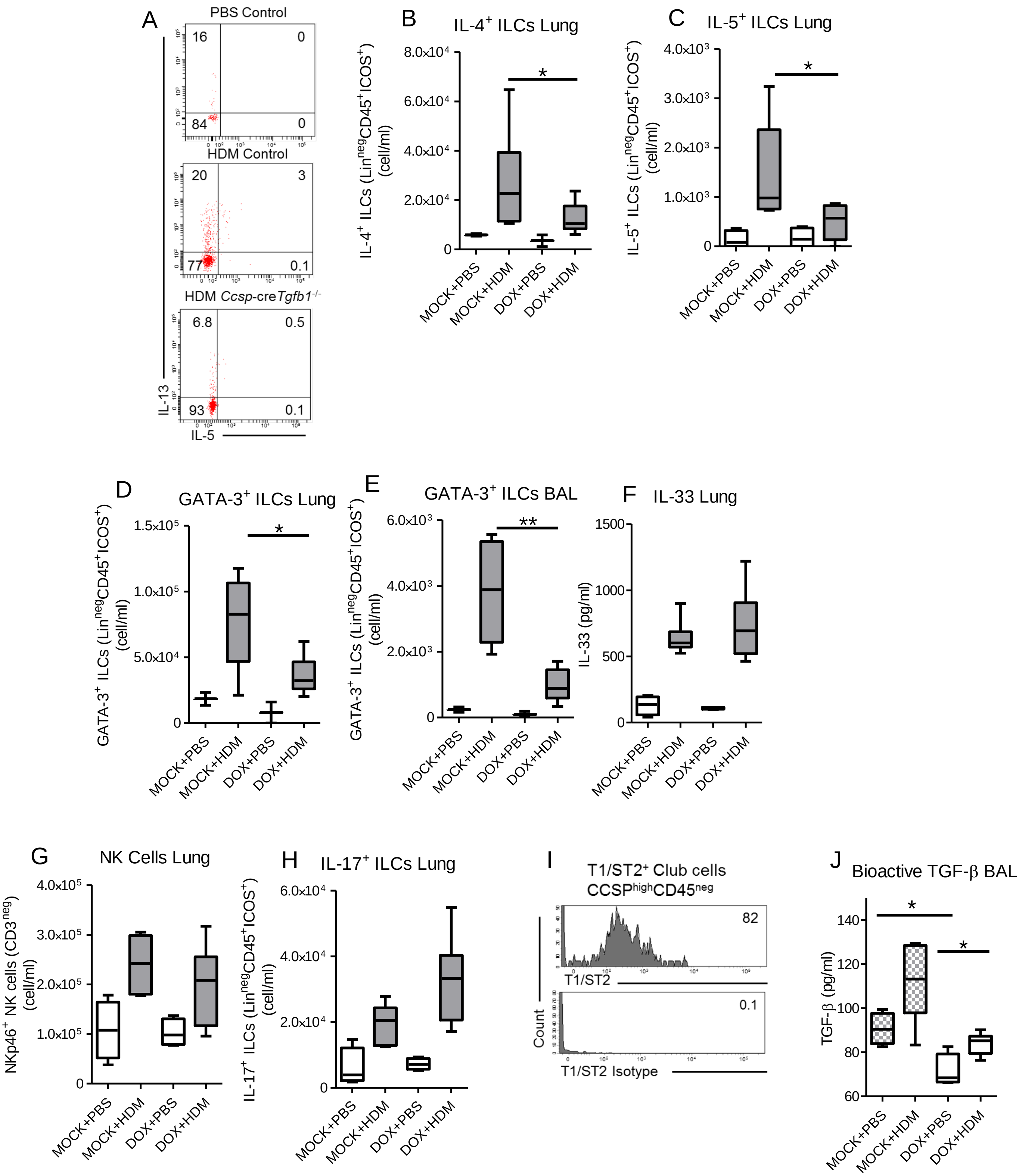
A
PBS Control
HDM Control
HDM Ccsp-cre Tgfb1-/-
IL-13
IL-5
B
IL-4+ ILCs Lung
IL-4+ ILCs (LinnegCD45+ICOS+) (cell/ml)
C
IL-5+ ILCs Lung
IL-5+ ILCs (LinnegCD45+ICOS+) (cell/ml)
D
GATA-3+ ILCs Lung
GATA-3+ ILCs (LinnegCD45+ICOS+) (cell/ml)
E
GATA-3+ ILCs BAL
GATA-3+ ILCs (LinnegCD45+ICOS+) (cell/ml)
F
IL-33 Lung
IL-33 (pg/ml)
G
NK Cells Lung
NKp46+ NK cells (CD3neg) (cell/ml)
H
IL-17+ ILCs Lung
IL-17+ ILCs (LinnegCD45+ICOS+) (cell/ml)
I
T1/ST2+ Club cells
CCSPhighCD45neg
T1/ST2
T1/ST2 Isotype
Count
J
Bioactive TGF-β BAL
TGF-β (pg/ml)
MOCK+PBS
MOCK+HDM
DOX+PBS
DOX+HDM

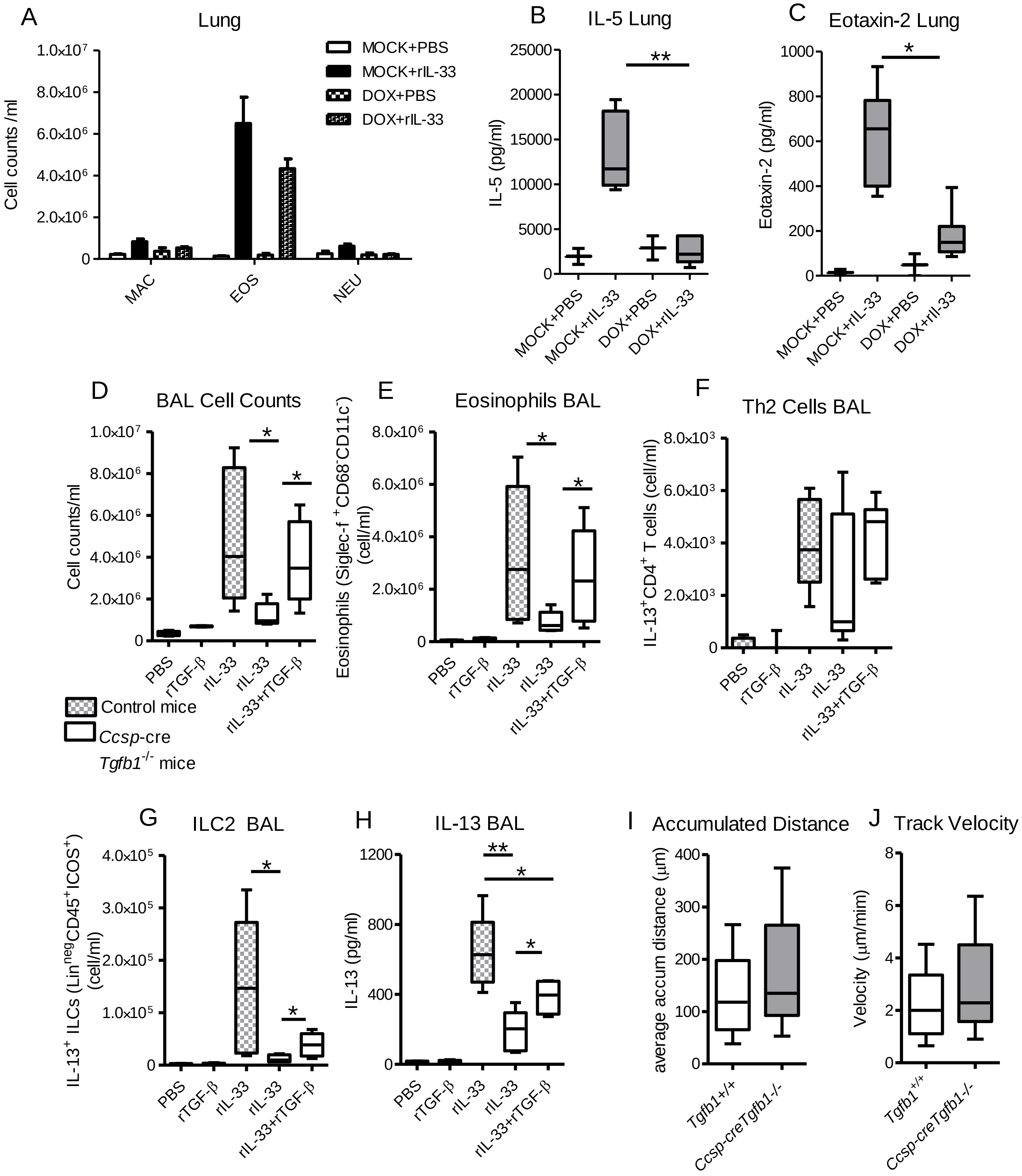

A
Lung
Cell counts /ml
MOCK+PBS
MOCK+rIL-33
DOX+PBS
DOX+rIL-33
MAC
EOS
NEU
B
IL-5 Lung
IL-5 (pg/ml)
**
C
Eotaxin-2 Lung
Eotaxin-2 (pg/ml)
*
D
BAL Cell Counts
Cell counts/ml
Control mice
Ccsp-cre Tgfb1-/- mice
E
Eosinophils BAL
Eosinophils (Siglec-f +CD68-CD11c-) (cell/ml)
F
Th2 Cells BAL
IL-13+CD4+ T cells (cell/ml)
G
ILC2 BAL
IL-13+ ILCs (LinnegCD45+ICOS+) (cell/ml)
H
IL-13 BAL
IL-13 (pg/ml)
PBS
rTGF-β
rIL-33
rIL-33+rTGF-β
I
Accumulated Distance
average accum distance (μm)
J
Track Velocity
Velocity (μm/mim)
Tgfb1+/+
Ccsp-creTgfb1-/-
