# Supplementary material for: Pulmonary Epithelial Cell-Derived Cytokine TGF-β1 Is a Critical Cofactor for Enhanced Innate Lymphoid Cell Function
Source: Immunity. 2015 Nov 17;43(5):945–58. doi: 10.1016/j.immuni.2015.10.012 (PMC4658339; doi:10.1016/j.immuni.2015.10.012)
Supplement: Document S2. Article plus Supplemental Information [file mmc2.pdf]

Immunity

Article

Pulmonary Epithelial Cell-Derived Cytokine TGF-β1 Is a Critical Cofactor for Enhanced Innate Lymphoid Cell Function

Graphical Abstract

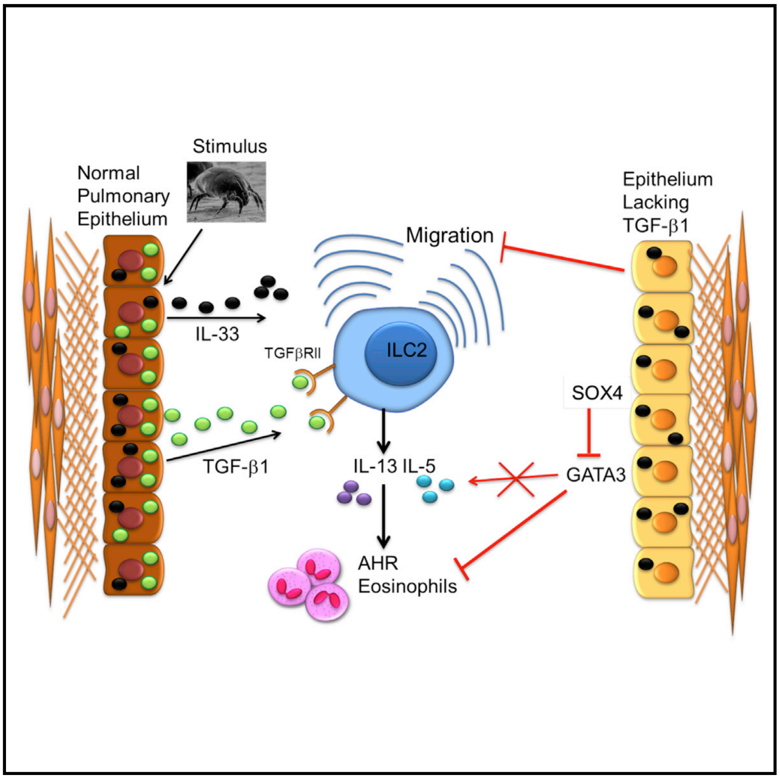

Highlights

- Epithelial-derived TGF-β drives pulmonary response to allergen
- Epithelial TGF-β promotes GATA3-driven cytokine production by suppression of Sox4
- TGF-β is chemoactive for innate lymphoid cells that express TGF-βRII
- The local pulmonary environmental niche defines the nature of immune reactions

Authors

Laura Denney, Adam J. Byrne, Thomas J. Shea, ..., Simone A. Walker, Lisa G. Gregory, Clare M. Lloyd

Correspondence

c.lloyd@imperial.ac.uk

In Brief

The role of epithelial-derived TGF-β in the pathogenesis of asthma is unknown. Lloyd and colleagues demonstrate that epithelial TGF-β is essential for the generation of the pulmonary allergic response to allergen by acting as a chemoactive factor for innate lymphoid type 2 cells and promoting GATA3-induced cytokine expression.

Denney et al., 2015, Immunity *43*, 945–958
November 17, 2015 ©2015 The Authors
http://dx.doi.org/10.1016/j.immuni.2015.10.012

CellPress

Pulmonary Epithelial Cell-Derived Cytokine TGF-β1 Is a Critical Cofactor for Enhanced Innate Lymphoid Cell Function

Laura Denney,[1] Adam J. Byrne,[1] Thomas J. Shea,[1] James S. Buckley,[1] James E. Pease,[1] Gaelle M.F. Herledan,[1] Simone A. Walker,[1] Lisa G. Gregory,[1] and Clare M. Lloyd[1,]*
[1]Inflammation, Repair & Development, National Heart and Lung Institute, Imperial College London, London SW7 2AZ UK
*Correspondence: c.lloyd@imperial.ac.uk
http://dx.doi.org/10.1016/j.immuni.2015.10.012
This is an open access article under the CC BY license (http://creativecommons.org/licenses/by/4.0/).

SUMMARY

Epithelial cells orchestrate pulmonary homeostasis and pathogen defense and play a crucial role in the initiation of allergic immune responses. Maintaining the balance between homeostasis and inappropriate immune activation and associated pathology is particularly complex at mucosal sites that are exposed to billions of potentially antigenic particles daily. We demonstrated that epithelial cell-derived cytokine TGF-β had a central role in the generation of the pulmonary immune response. Mice that specifically lacked epithelial cell-derived TGF-β1 displayed a reduction in type 2 innate lymphoid cells (ILCs), resulting in suppression of interleukin-13 and hallmark features of the allergic response including airway hyperreactivity. ILCs in the airway lumen were primed to respond to TGF-β by expressing the receptor TGF-βRII and ILC chemoactivity was enhanced by TGF-β. These data demonstrate that resident epithelial cells instruct immune cells, highlighting the central role of the local environmental niche in defining the nature and magnitude of immune reactions.

INTRODUCTION

Regulation of innate immunity is essential for maintenance of immune homeostasis, preventing inappropriate immune activation and associated pathology. Maintaining this balance is particularly complex at mucosal sites, which are exposed to billions of potentially antigenic particles daily. For example, the pulmonary immune system must be poised to respond quickly and efficiently to inhaled pathogens such as respiratory viruses while ignoring innocuous material from the inhaled environment such as dust, pollen, and animal dander. Thus, an intricate network of regulatory pathways is employed to facilitate maintenance of homeostasis. Although regulatory T cells and interleukin-10 (IL-10) are an essential component of this system, the role of transforming growth factor-β (TGF-β) is less clear. TGF-β promotes the expression of the transcription factor FOXP3, thereby facilitating generation of $CD4^+CD25^+$ regulatory T (Treg) cells that are able to inhibit allergic airway disease (Chen et al., 2003; Kearley et al., 2005). Conversely, TGF-β also drives lineage specificity in effector T cell subsets. Induction of the transcription factor RORγT-dependent differentiation pathway in $CD4^+$ T cells can result in either T helper 17 (Th17) or Treg cells depending on concomitant expression of maturation factors such as IL-6, IL-21, retinoic acid, IL-23, and IL-10 (Travis and Sheppard, 2014). Similarly, a combination of TGF-β, IL-25, and IL-4 drives Th9 cell generation (Dardalhon et al., 2008; Jones et al., 2012). The collective activity of TGF-β and IL-10 ensures control of inflammatory responses and promotes effective immunity against pathogens while restricting excessive immunopathology to self or inhaled particles (Li and Flavell, 2008).

TGF-β is expressed constitutively by a wide variety of leukocytes and stromal cells within the lung, including alveolar macrophages, smooth muscle cells, fibroblasts, and the epithelium (de Boer et al., 1998; Sullivan et al., 2009). Indeed, the lung epithelium plays an active role in directing the immune response to both pathogens and allergens. Manipulation of epithelial genes to promote TGF-β signaling results in an exacerbation of house dust mite (HDM)-induced pathology (Gregory et al., 2010) and loss of tolerance to inhaled ovalbumin (Gregory et al., 2013). Epithelial cells can release chemokines and cytokines including IL-6, TNF-α, IFN-α, IFN-β, GM-CSF, MIP-1α (CCL3), and MCP-1 (CCL2) upon antigen stimulation, culminating in cell recruitment and activation (Lambrecht and Hammad, 2012; Vareille et al., 2011). In an allergic context, epithelial cell secretion of the cytokines IL-25, IL-33, and TSLP promote Th2 cell and innate lymphoid type 2 cell (ILC2) recruitment (Licona-Limón et al., 2013).

Expression of TGF-β is increased in the lung after both viral and allergen challenge (Gibbs et al., 2009; Kariyawasam et al., 2009; Schultz-Cherry and Hinshaw, 1996). Moreover, SNPs in the promoter and coding regions of TGF-β (which result in increased gene expression) have been linked to asthma susceptibility (Li et al., 2007; Silverman et al., 2004). The crucial role TGF-β plays in maintaining peripheral tolerance has long been established, with global genetic deletion of TGF-β resulting in early death from multi-organ inflammation (Shull et al., 1992). Interestingly, targeted deletion of TGF-β signaling in $CD4^+$ T cells results specifically in inflammation at mucosal sites, including the airways (Li and Flavell, 2008). We and others have previously determined that systemic neutralization of

CrossMark

TGF-β via antibodies has variable effects on lung remodeling, inflammation, and airway hyperactivity (AHR), depending on the route of allergen exposure (Fattouh et al., 2008; McMillan et al., 2005).

It has been postulated that asthma results from a loss of tolerance to harmless airborne particles; we hypothesized that a local imbalance of TGF-β in the lung might modulate this loss of tolerance. In order to investigate the specific role of epithelial-derived TGF-β in directing the pulmonary immune response to inhaled allergen, we generated mice with a conditional deletion of *Tgfb1* in epithelial cells. Mice lacking epithelial-derived TGF-β1 displayed no baseline immune defects but were protected from the effects of allergen exposure, exhibiting diminished airway inflammation and improved lung function. Although pulmonary IL-13$^+$ Th2 cells were unaffected, the frequency of IL-13$^+$ ILC2s was significantly reduced. We found that ILCs expressed TGF-βRII and moreover, exposure of airway ILCs to TGF-β increased cell chemoactivity. This novel interaction between ILCs and TGF-β derived from the lung epithelium is a crucial pathway leading to the generation of early allergic immune responses and reinforces the concept that resident tissue stromal cells are key facilitators in the inception of local mucosal immunity.

RESULTS

Epithelial-Derived TGF-β Is Critical for the Development of Allergic Immunity

TGF-β is secreted by many cells within the lung and is critical for a range of immune functions, including the generation and regulation of effector T cell subsets, which have key roles at mucosal surfaces. In order to determine how pulmonary epithelial-derived TGF-β directs lung immunity, we generated mice lacking TGF-β specifically in the bronchial epithelium (*Ccsp*-cre*Tgfb1*$^{-/-}$) and assessed the effect of allergen exposure. Deletion of TGF-β in the airway epithelium was achieved by crossing mice expressing a TGF-β1 gene containing loxP sites flanking exon 6 with club (previously Clara) (Winkelmann and Noack, 2010) cell secretory protein (CCSP-$_{rtTA/tetO}$-Cre) transgenic mice (Figure S1A; Azhar et al., 2009; Perl et al., 2009). This inducible system allows the lungs to develop normally until administration of doxycycline (DOX) in adulthood, thereby avoiding potentially adverse effects of TGF-β deletion on lung organogenesis and development.

Cre recombinase expression in the lung was restricted to epithelial cells of the conducting airways and was detectable within 48 hr of intraperitoneal dosing with DOX with very little non-specific expression in other lung structural or resident cells (Figure S1B). 72 hr after treatment with DOX, TGF-β1 was deleted in bronchiolar epithelial cells in transgenic mice whereas expression was unaffected in littermate controls (Figure S1C). Additionally, we sorted club epithelial cells based on surface expression of CCSP. mRNA levels of TGF-β1 were unaffected in the CCSP$^-$CD45$^+$ cell fraction of *Ccsp*-cre*Tgfb1*$^{-/-}$ mice compared to control mice but were greatly decreased in the club cell fraction (CCSP$^+$CD45$^-$), demonstrating the specificity of TGF-β1 deletion in epithelial cells of the conducting airways (Figure S1D). Global and T-cell-specific TGF-β knockout mice develop lymphoid proliferative disease and widespread inflammation at baseline (Kulkarni et al., 1993; Li et al., 2006). In contrast, *Ccsp*-cre*Tgfb1*$^{-/-}$ mice exhibited no significant inflammation in the blood, spleen, bone marrow, or mediastinal lymph nodes (Figures S1E–S1H). Functional TGF-β is vital for suppression of inappropriate immune responses to innocuous airborne particles/antigens, such as dust and dander (Fattouh et al., 2008). Given the importance of the pulmonary epithelial barrier in defense against these antigens, we hypothesized that epithelial-derived TGF-β would be important in the regulation of immune responses to inhaled allergens. Mice were pre-treated with DOX or vehicle (mock) 72 hr prior to intra-nasal administration of the common aeroallergen house dust mite extract (HDM) for 3 weeks in a continuous dosing protocol. Ablated epithelial expression of TGF-β was maintained after 3 weeks of HDM exposure in *Ccsp*-cre*Tgfb1*$^{-/-}$ mice (Figure 1A). We observed no compensatory increase in expression of *Tgfb2* or *Tgfb3* in the lung tissue of mice lacking epithelial-derived TGF-β1 expression (Figure S2A). Because doxycycline is a potent antibiotic and long-term exposure has previously been shown to alter lung structure, we conducted parallel experiments in littermate control mice with homozygous *Tgfb1*$^{fl/fl}$ but lacking either *CCSP*-$_{rtTA}$ or $_{tetO}$-Cre. DOX administration alone did not significantly affect the allergic phenotype; HDM exposure promoted similar levels of airway hyperreactivity (AHR), inflammation, and eosinophilia in both DOX- and mock-treated littermate control groups (Figures S2B–S2F). These results indicate that mice deficient in epithelial-derived TGF-β are immunologically normal under homeostatic conditions.

In contrast, *Ccsp*-cre*Tgfb1*$^{-/-}$ mice had significantly reduced AHR in response to HDM exposure with ameliorated airway resistance and compliance compared to mock-treated mice (Figures 1B and S2G). Recruitment of cells to the airway lumen was also reduced in *Ccsp*-cre*Tgfb1*$^{-/-}$ mice (Figure 1C), although recruitment to the lung tissue was unaffected (Figure 1D). This was reflected in the similar levels of peribronchial inflammation as measured by histological scoring of lungs between *Ccsp*-cre*Tgfb1*$^{-/-}$ mice and mock-treated mice (Figures 1E and S2H). Specific epithelial deletion of TGF-β greatly diminished HDM-induced pulmonary eosinophilia, a hallmark feature of asthma (Figures 1F and S2I). This was accompanied by a decrease in the amount of the eosinophilic chemokine eotaxin-2 in the airways but not the lung (Figures 2A and 2B), as well as the eosinophil growth and survival factor IL-5, which was dramatically reduced in both the airways and lung tissue (Figures 2C and 2D). The effect seemed to be highly selective for eosinophils because the numbers of neutrophils and macrophages and the amounts of their associated chemoattractants were unaffected (Figures 1F and S3A–S3C). The lack of epithelial-derived TGF-β resulted in a small basal increase in pulmonary eosinophil numbers in these mice (Figure 1F), which was not attributable to DOX treatment because the same phenotype was not observed in incomplete transgenic littermate controls that lack the genetic machinery to excise epithelial-derived TGF-β (Figure S2F). Thus, epithelial-derived TGF-β is a crucial mediator of allergen-driven eosinophil recruitment to the lung.

IL-4 is classically involved in immunoglobulin class switching for the generation of allergen-specific immunoglobulin E (IgE) and is characteristic of atopic asthma. We determined that the lack of epithelial-derived TGF-β completely abrogated the IL-4 response in the lung (Figure 2E) and led to diminished total and

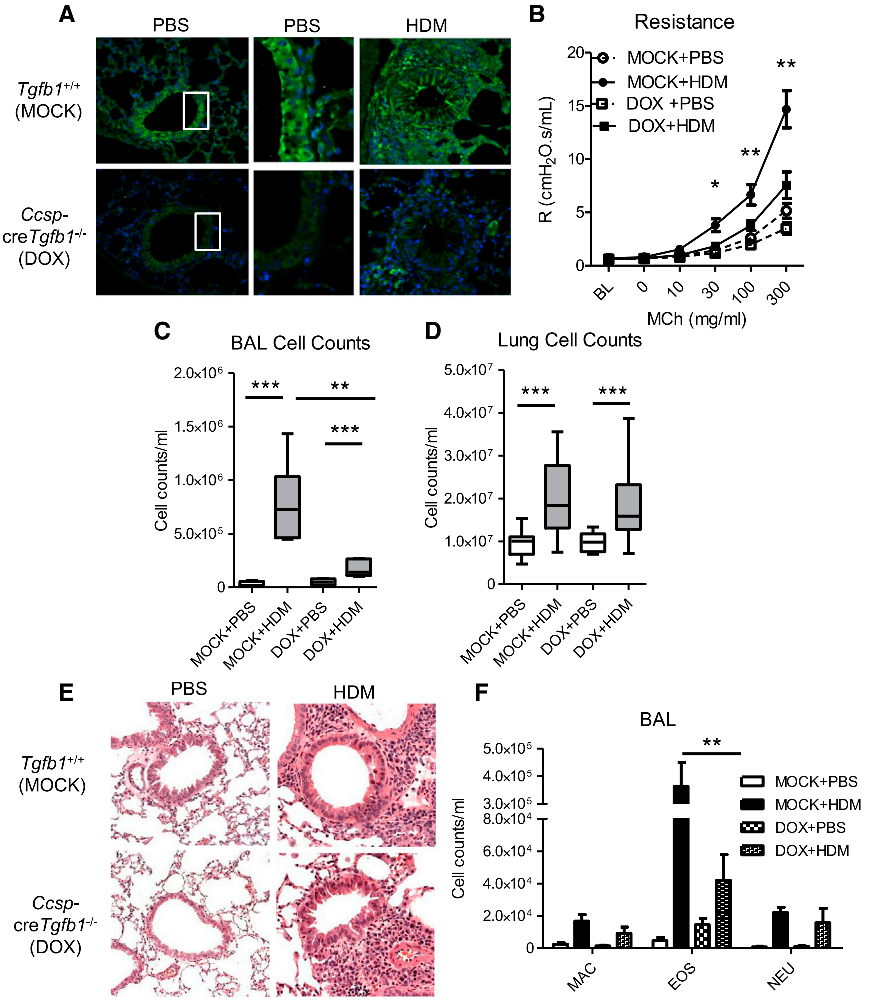

Figure 1. Mice Lacking Epithelial-Derived TGF-β Have Reduced Airway Hyperreactivity, Airway Inflammation, and Eosinophilia

(A) TGF-β1 expression in the epithelium of *Ccsp*-cre*Tgfb1*$^{-/-}$ (DOX)- and *Tgfb1*$^{+/+}$ (mock)-treated mice after intranasal house dust mite (HDM) or PBS administration for 3 weeks. Original magnification 20×.

(B) Airway hyperreactivity measured by airway resistance to ascending methacholine concentration (baseline; BL).

(C and D) Cell counts in the (C) airways and (D) lung tissue.

(E) Hematoxylin and eosin-stained lung tissue after HDM administration.

(F) Numbers of macrophages (MAC), eosinophils (EOS), and neutrophils (NEU) in the airways.

Mann-Whitney *$p < 0.05$, **$p < 0.01$, and ***$p < 0.001$. Data shown are from one experiment representative of two independent experiments with a total of n = 10–12 mice per group. Box and whisker plots depict the median and IQR and minimum and maximum values. Line graphs and bar charts are expressed as mean ± SEM. See also Figures S1 and S2.

allergen-specific IgE (Figures 2F and 2G). TGF-β is known to induce IgA production and global TGF-β-deficient mice are unable to mount an effective IgA response (van Ginkel et al., 1999). However, there was no effect on local pulmonary IgA levels in mice specifically lacking epithelial-derived TGF-β (Figures 2H and S3D), indicating that TGF-β derived from the airway epithelium is necessary for the generation of IL-4 in response to allergen challenge and subsequent production of IgE.

IL-13, the prototypic type 2 cytokine, is a critical driver of AHR and coincident with the diminished AHR, we found that IL-13 protein was reduced both in the lung tissue and airways of HDM-exposed *Ccsp*-cre*Tgfb1*$^{-/-}$ mice (Figures 2I and 2J). There was also a concomitant reduction in pulmonary *Il13* mRNA levels in these mice (Figure 2K), implying that epithelial-derived TGF-β drives type 2 immune responses and the ensuing AHR after HDM exposure.

Epithelial-Derived TGF-β Specifically Affects Accumulation of ILC2s rather than IL-13$^+$ Th2 Cells

TGF-β is a crucial controller of T cell lineage differentiation. Because we observed a reduction in type 2 immunity and because TGF-β has also been shown to suppress Th2 cell differentiation (Travis and Sheppard, 2014), we enumerated these cells in lungs from *Ccsp*-cre*Tgfb1*$^{-/-}$ and control mice. As expected, in mock-treated mice, HDM exposure resulted in an increase in the frequency of Th2 (CD3$^+$CD4$^+$IL-13$^+$) cells (gating strategy shown in Figure S3E). However, despite the reduction in pulmonary IL-13 concentrations in *Ccsp*-cre*Tgfb1*$^{-/-}$ mice, the numbers of IL-13$^+$ Th2 cells were comparable in both the lung and BAL from *Ccsp*-cre*Tgfb1*$^{-/-}$ and control mice (Figures 3A and 3B). We did, however, observe a decrease in the number of IL-4$^+$ T cells (Figure S3F). The observed reduction in IL-4$^+$ T cells and no change in IL-13$^+$ T cells in *Ccsp*-cre*Tgfb1*$^{-/-}$ mice is probably due to the complex and differential interactions between GATA-3 and the IL-4 and IL-5/13 promoter sites (Van Stry and Bix, 2011).

Similarly, the absence of epithelial-derived TGF-β had no effect on development of either Th17 or Th1 cells (Figures 3C, 3D, S3G, and S3H) or the production of cytokines IL-17 or IFN-γ (Figures S3I and S3J), which might drive part of the residual inflammation observed in the lungs of *Ccsp*-cre*Tgfb1*$^{-/-}$ mice despite reduced Th2 cytokines. The numbers of IL-10$^+$ Treg and Foxp3$^+$ Treg cells were also not different in lungs from *Ccsp*-cre*Tgfb1*$^{-/-}$ mice (Figures 3E and 3F). Thus, although lymphocyte-derived TGF-β has been reported to have profound effects on Th cell differentiation in vitro and in other in vivo model systems, it appears that local epithelial-derived TGF-β makes little contribution to directing Th cell subset generation at the pulmonary mucosa.

ILCs are postulated to be an important source of IL-13 in both allergic and viral responses—particularly contributing to the development of AHR (Kim et al., 2012). Therefore, we

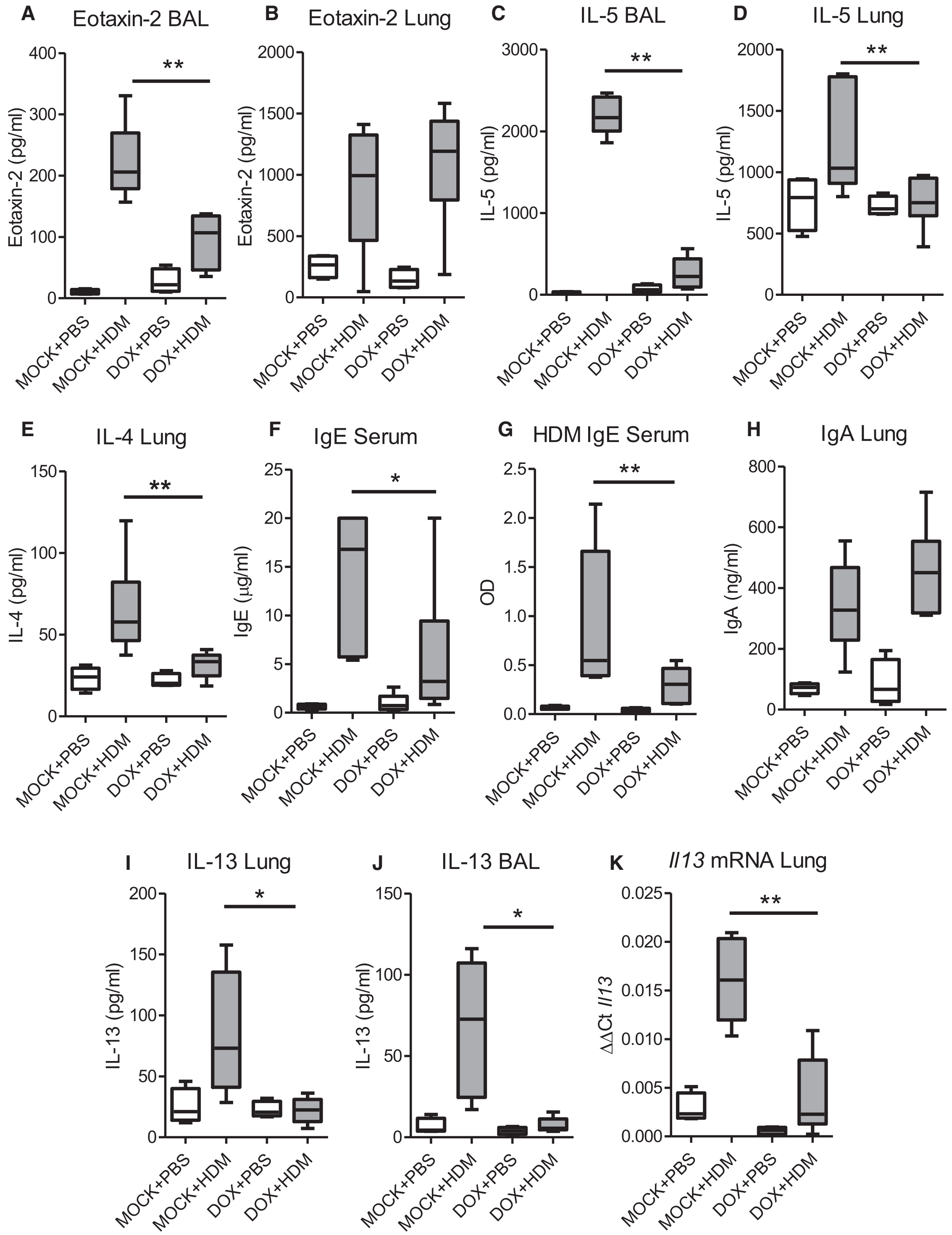

Figure 2. Epithelial-Derived TGF-β Is Necessary for the Generation of Hallmark Features of Allergic Airways Disease

(A and B) Levels of eotaxin-2 in the (A) BAL and (B) lung of *Ccsp*-cre*Tgfb1*$^{-/-}$ (DOX) and *Tgfb1*$^{+/+}$ (mock) mice after intranasal house dust mite (HDM) or PBS administration.

(C and D) IL-5 levels in the (C) BAL and (D) lung.

(E) Concentration of IL-4 in lung tissue.

(F and G) Serum levels of (F) IgE and (G) HDM-specific IgE.

(H) IgA in lung tissue.

(legend continued on next page)

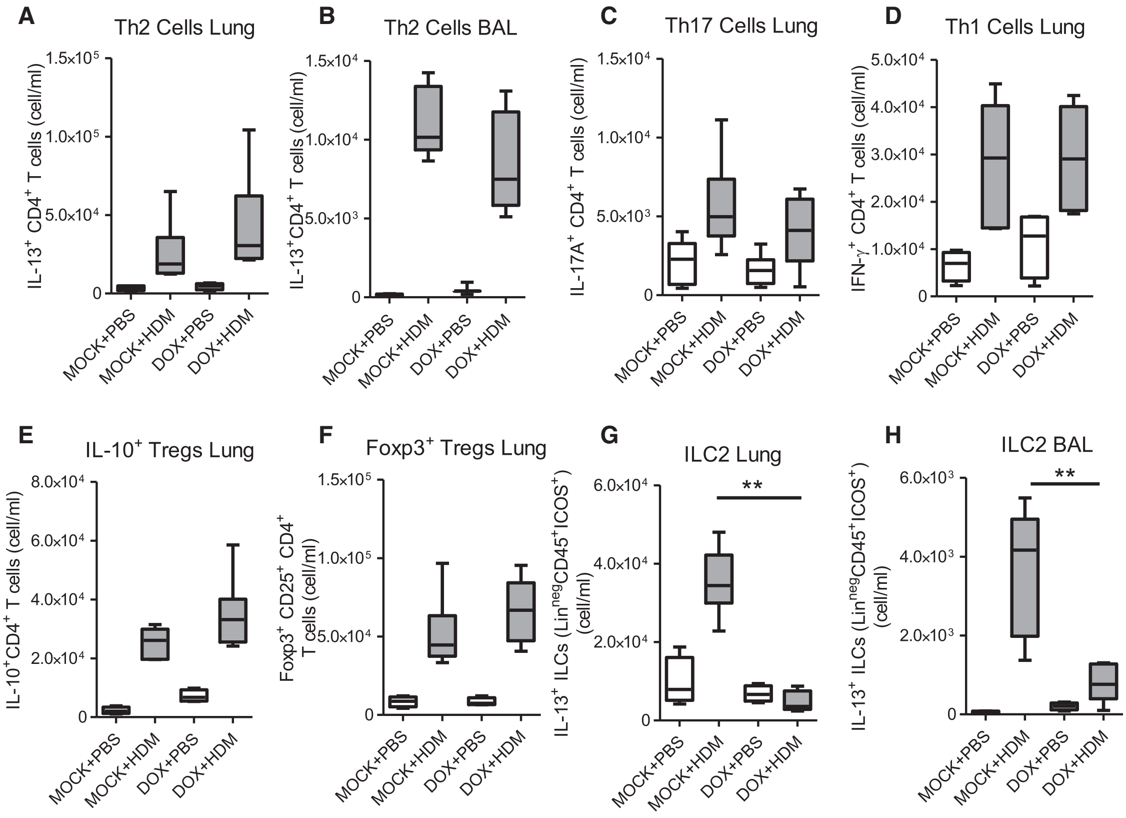

Figure 3. IL-13^{+} ILCs rather than T Effector Cell Subsets Are Preferentially Reduced in *Ccsp*-cre*Tgfb1*$^{-/-}$ Mice

(A and B) Frequencies of Th2 cells in the (A) lung tissue and (B) airways of *Ccsp*-cre*Tgfb1*$^{-/-}$ (DOX)- and *Tgfb1*$^{+/+}$ (mock)-treated mice after intranasal house dust mite (HDM) or PBS administration.

(C–F) Numbers of (C) Th17 cells, (D) Th1 cells, (E) IL-10^{+} Treg cells, and (F) FOXP3^{+} Treg cells in the lung.

(G and H) Frequencies of IL-13^{+}lineage^{-}CD45^{+}ICOS^{+} ILCs in the (G) lung and (H) airways.

Mann-Whitney **$p < 0.01$. Data shown are from one experiment representative of two independent experiments with a total of n = 10–12 mice per group. Box and whisker plots depict the median and IQR and minimum and maximum values. See also Figures S3–S5.

enumerated the IL-13^{+} ILC2 population defined here as IL-13^{+} Lin^{-}ICOS^{+}CD45^{+} cells (gating strategy outlined in Figures S4A and S4B). Further analysis of these IL-13^{+} ILCs shows that these cells strongly expressed CD127, Thy1, and ST2 (but not CD3, TER-119, CD11b, GR1, CD45R, CD4, CD5, CD8, NK1.1, CD11c, TCR-β, or TCR-γδ) (Figures S4C and S4D). Although epithelial-derived TGF-β had no effect on the numbers of IL-13^{+} T cells, the numbers of IL-13^{+} ILC2s were significantly reduced in *Ccsp*-cre*Tgfb1*$^{-/-}$ mice compared to control mice in both the lungs and airways (Figures 3G, 3H, and S5A) as were both IL-4^{+} and IL-5^{+} ILCs (Figures S5B and S5C), correlating with the decreased concentrations of these cytokines measured in the lung. Total ILC2s (including non-activated i.e., not expressing cytokine but expressing the ILC2 transcription factor GATA-3) were also reduced in lung and bronchoalveolar lavage (BAL) of allergen-treated *Ccsp*-cre*Tgfb1*$^{-/-}$ mice (Figures S5D and S5E), despite concentrations of the ILC-inducing cytokine IL-33 being equivalent in control and *Ccsp*-cre*Tgfb1*$^{-/-}$ mice (Figure S5F). This affect was restricted to the ILC2 population, as shown by the fact that numbers of NK cells and ILC3s (IL-17^{+}) were comparable in *Ccsp*-cre*Tgfb1*$^{-/-}$ and control mice (Figures S5G and S5H).

These findings establish that epithelial-derived TGF-β has a highly cell-selective effect and rather than contributing here to the differentiation of effector T cell subsets, it appears to be critical for the accumulation of type 2 ILCs in the lung and airways after allergen exposure. Thus, epithelial-derived TGF-β is necessary for the development of key hallmark features of allergic airways disease including increased ILC2s, type 2 cytokine induction, AHR, eosinophilia, and IgE.

Epithelial-Derived TGF-β Acts in Concert with IL-33 to Facilitate Mucosal Inflammation

ILC2s are induced by a trio of epithelial cytokines of variable potency: IL-33, IL-25, and TSLP (Barlow et al., 2013). Because the IL-13^{+} ILC2 population appears to be strongly influenced

(I and J) Levels of IL-13 in the (I) lung tissue and (J) BAL fluid.

(K) *Il13* mRNA levels in lung tissue.

Mann-Whitney *$p < 0.05$ and **$p < 0.01$. Data shown are from one experiment representative of two independent experiments with a total of n = 10–12 mice per group. Box and whisker plots depict the median and IQR and minimum and maximum values. See also Figure S3.

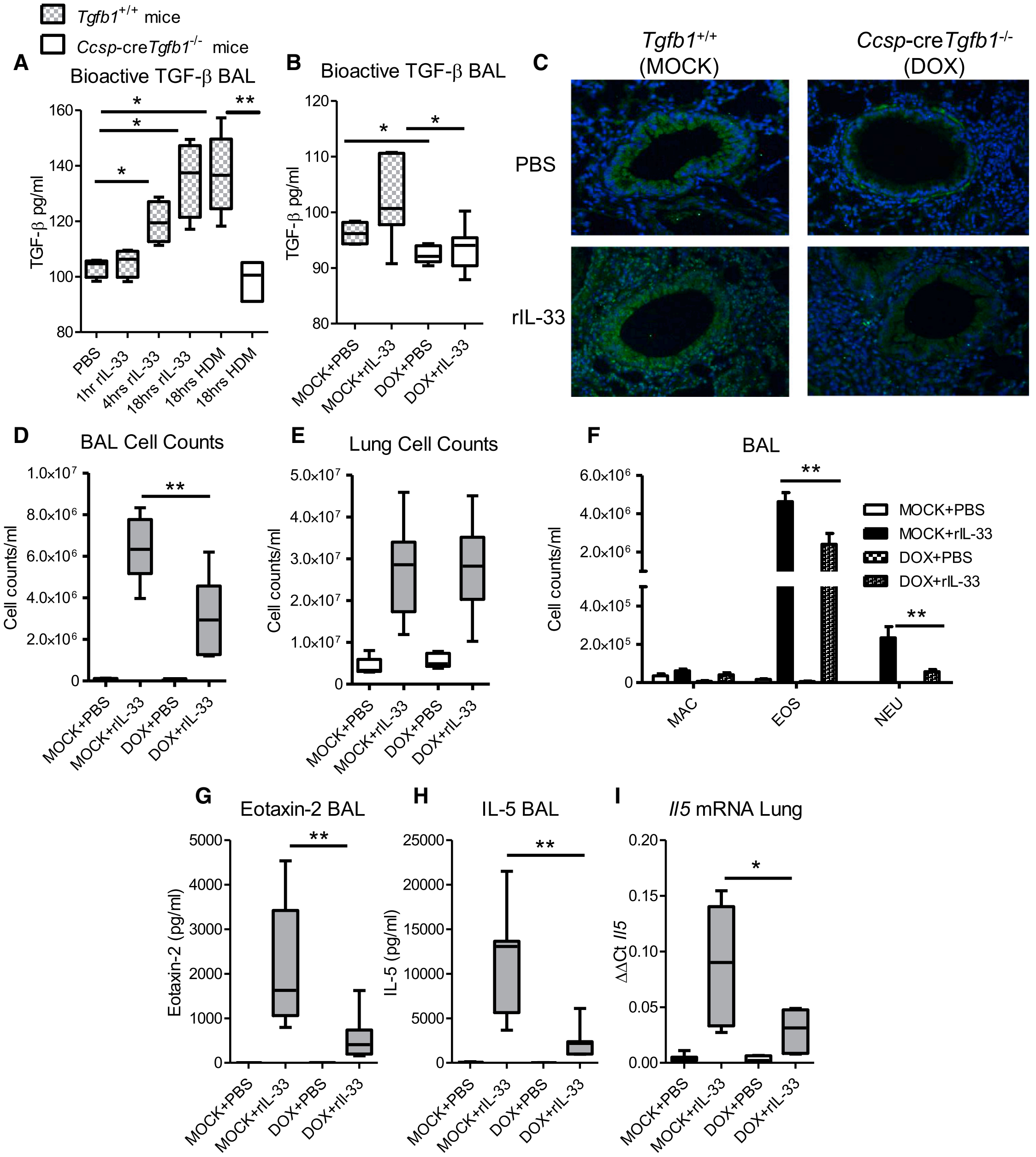

Figure 4. Epithelial-Derived TGF-β Enhances IL-33-Mediated Inflammation

(A) Levels of TGF-β in the BAL of control (*Tgfb1*$^{+/+}$) and *Ccsp*-cre*Tgfb1*$^{-/-}$ mice treated with a single dose of either rIL-33 or HDM as measured by TGF-β bioassay.

(B) TGF-β in the BAL of *Ccsp*-cre*Tgfb1*$^{-/-}$ (DOX) and *Tgfb1*$^{+/+}$ (mock) mice after 1 week of rIL-33 (or PBS) administration.

(C) TGF-β1 expression in the epithelium in DOX- and mock-treated mice. Original magnification 20×.

(D and E) Cell counts in the (D) airways and (E) lung tissue.

(F) Numbers of macrophages (MAC), eosinophils (EOS), and neutrophils (NEU) in the airways.

(G–I) Levels of (G) Eotaxin-2 and (H) IL-5 in the BAL and (I) *Il5* mRNA levels in the lung tissue.

Mann-Whitney *p < 0.05 and **p < 0.01. Data shown are from one experiment representative of two independent experiments with a total of n = 10–12 mice per group. Box and whisker plots depict the median and IQR and minimum and maximum values. Bar charts are expressed as mean ± SEM. See also Figures S5 and S6.

by epithelial-derived TGF-β, we further investigated the relationship between ILC-inducing cytokines and TGF-β by utilizing a week-long model of recombinant IL-33 (rIL-33) administration to induce substantial ILC2 numbers in the absence of an antigen-specific Th2 cell response (as observed in response to allergen exposure over a 3 week period). To determine whether IL-33 directly influenced TGF-β release, we first administered a single dose of murine rIL-33 (1 μg) intranasally to control (*Tgfb1*$^{+/+}$) mice and BAL fluid was recovered 1, 4, and 18 hr later (Figure 4A). rIL-33 administration induced a rapid release of

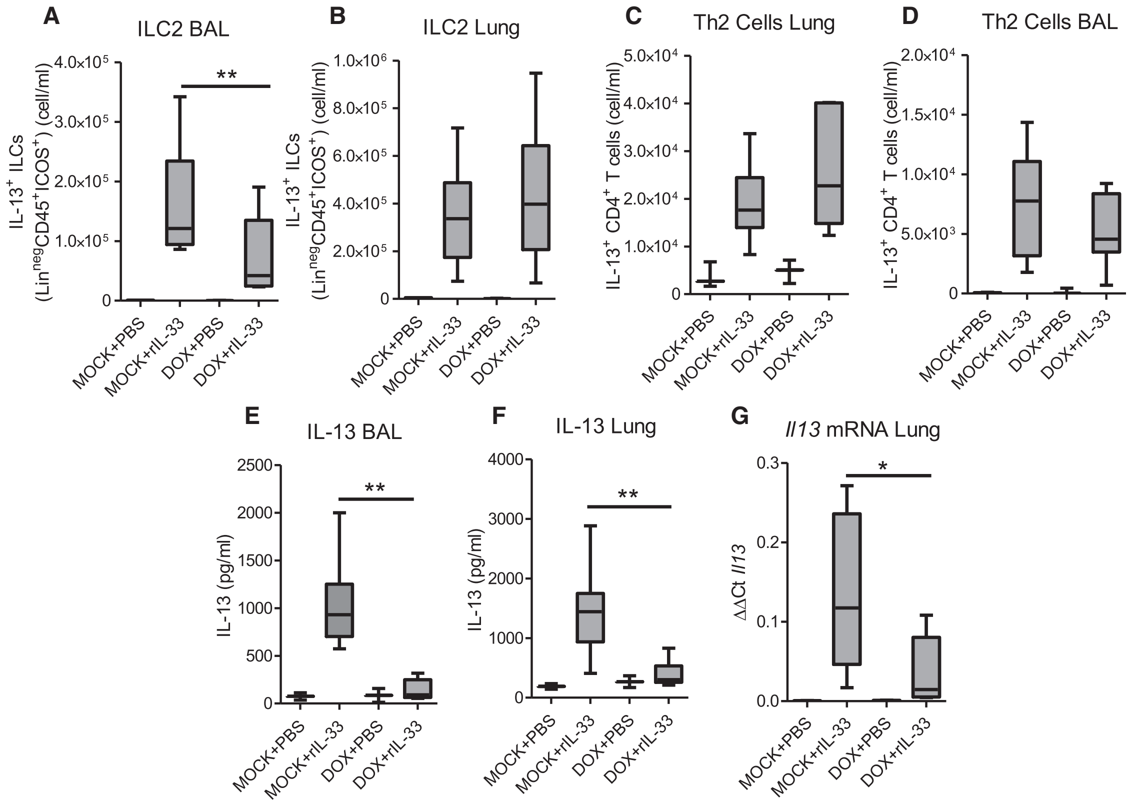

Figure 5. Epithelial-Derived TGF-β Enhances IL-13$^+$ ILC Responses during IL-33-Driven Inflammation

(A and B) Frequencies of IL-13$^+$lineage$^-$CD45$^+$ICOS$^+$ ILCs in the (A) airways and (B) lung tissue in *Ccsp*-cre*Tgfb1*$^{-/-}$ (DOX) and *Tgfb1*$^{+/+}$ (mock) mice after rIL-33 (or PBS) administration.

(C and D) Th2 cells in the (C) lung tissue and (D) airways.

(E and F) Levels of IL-13 in the (E) BAL fluid and (F) lung tissue.

(G) *Il13* mRNA levels in lung tissue.

Mann-Whitney *$p < 0.05$, **$p < 0.01$. Data shown are from one experiment representative of two independent experiments with a total of n = 10–12 mice per group. Box and whisker plots depict the median and IQR and minimum and maximum values.

active TGF-β into the airway lumen. Similarly, a single dose of HDM resulted in comparable TGF-β release (Figure 4A). Interestingly, this enhanced production of bioactive TGF-β after HDM stimulation was not observed in mice specifically lacking epithelial-derived TGF-β (Figure 4A, white box). This indicated that IL-33 specifically enhanced release of TGF-β from the lung epithelium, which expresses the IL-33 receptor ST2 (Figure S5I), since there is no compensatory release of TGF-β in *Ccsp*-cre*Tgfb1*$^{-/-}$ mice that still contained TGF-β-sufficient leukocytes and other pulmonary cells. However, ST2-deficient mice exposed to 3 weeks of HDM display equivalent levels of TGF-β release (data not shown), indicating that functional IL-33 signaling was not absolutely required for release of TGF-β. The secretion of epithelial-derived TGF-β was maintained after multiple doses of both rIL-33 (Figure 4B) and HDM (Figure S5J). Immuno-histochemical staining of lung sections also localized TGF-β to the bronchial epithelium after rIL-33 administration (Figure 4C). As expected, in *Ccsp*-cre*Tgfb1*$^{-/-}$ mice expression of TGF-β in the epithelium was absent after rIL-33 treatment.

Pulmonary delivery of IL-33 in vivo is associated with type 2 inflammation mediated predominantly by ILC2s (Barlow et al., 2013; Walker and McKenzie, 2012). A lack of epithelial-derived TGF-β resulted in a significantly reduced cellular infiltration to the airway lumen rather than the lung tissue (Figures 4D, 4E, and S6A). Differential cell counts revealed that the reduction in IL-33-induced BAL inflammation was due to decreased recruitment of both eosinophils and neutrophils in *Ccsp*-cre*Tgfb1*$^{-/-}$ mice (Figure 4F). The reduction in BAL eosinophilia in the *Ccsp*-cre*Tgfb1*$^{-/-}$ mice was probably due to reduced eosinophil trafficking to the airways because lower levels of the key eosinophil chemoattractant eotaxin-2 and also IL-5 were detected in the BAL (Figures 4G and 4H). Similar findings were observed in the lung (Figure S6B and S6C). Lung *Il5* mRNA levels were also reduced in *Ccsp*-cre*Tgfb1*$^{-/-}$ mice (Figure 4I).

Mice that specifically lack TGF-β in their pulmonary epithelial cells had significantly reduced ILC2 numbers in the airway lumen after rIL-33 treatment (Figure 5A). In contrast, numbers of ILC2s and Th2 cells within the lung tissue were unaffected (Figures 5B and 5C). Th2 cells in the airway lumen also remained unchanged (Figure 5D). After rIL-33 administration, the IL-13 response was significantly decreased in the lungs and airways of *Ccsp*-cre*Tgfb1*$^{-/-}$ mice, at both protein and RNA levels (Figures 5E–5G). Administration of exogenous TGF-β to *Ccsp*-cre*Tgfb1*$^{-/-}$ mice restored the IL-33-induced increase in BAL inflammation

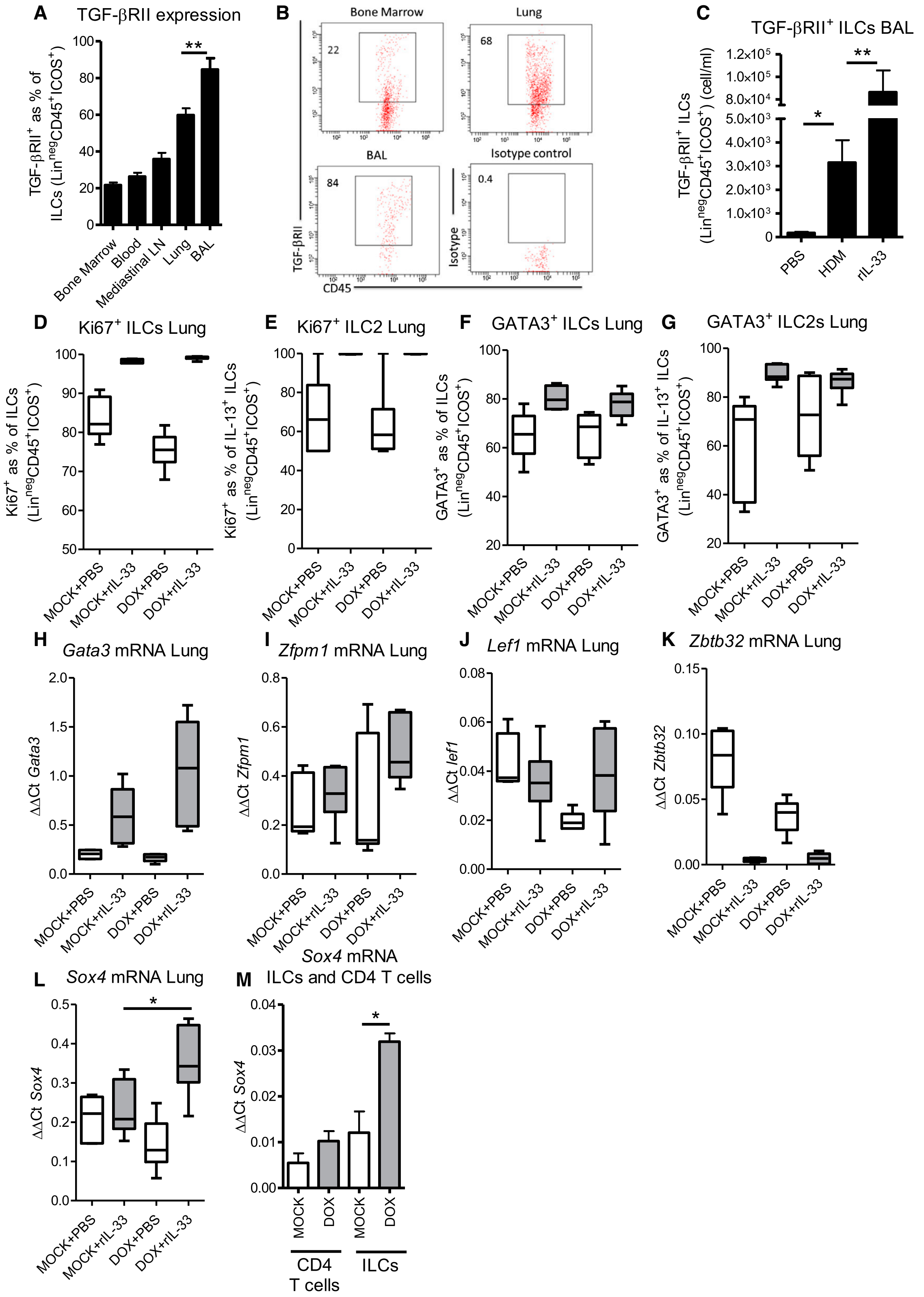

Figure 6. TGF-β Acts to Enhance ILC2 Activation

(A) TGF-βRII expression on ILCs defined as lineage⁻CD45⁺ICOS⁺ in different organs (lymph nodes [LN]), with resident ILC populations in naive control (*Tgfb1*$^{+/+}$) mice.

(legend continued on next page)

(Figure S6D). Airway eosinophilia and Th2 cell recruitment, which was reduced in *Ccsp*-cre*Tgfb1*$^{-/-}$ mice, was elevated to comparable levels as control IL-33-treated mice (Figures S6E and S6F). There was also a partial restoration of ILC2 numbers and IL-13 secretion in the *Ccsp*-cre*Tgfb1*$^{-/-}$ mice treated with rTGF-β (Figures S6G and S6H). The number of pulmonary ILCs and the levels of Th2 cytokines were 10-fold greater in the rIL-33 model of type 2 inflammation compared to that observed in the allergen-induced allergic airways disease model. However, whereas the magnitude of the immune response was substantially increased, the effect of epithelial-derived TGF-β was consistent in reducing IL-13^{+} ILC2 accumulation. Thus, TGF-β secreted from airway epithelial cells is fundamental for the inception of type 2 immunity after multiple stimuli.

Pulmonary IL-13^{+} ILC2s Are Primed to Respond to Epithelial-Derived TGF-β

Targeted deletion of TGF-β in the bronchial epithelium had a specific effect on IL-13^{+} ILC2 responses in the airways after exposure to either allergen or rIL-33. We measured expression of TGF-βRII on ILCs in different anatomical compartments of naive control mice to determine whether cells in the airways are specifically primed to respond to TGF-β. Indeed, we found a significantly higher percentage of ILCs in the airway lumen-expressed TGF-βRII compared to ILCs in the lung tissue, bone marrow, lung lymph nodes, or blood (Figures 6A and 6B), indicating that ILCs in the airway lumen are primed to respond to TGF-β secreted in response to inhaled environmental stimuli. Indeed, both HDM and rIL-33 resulted in enhanced accumulation of TGF-βRII-expressing ILCs in the airways (Figure 6C).

During manipulation of an inflammatory response, reduced accumulation of a particular cell type can occur as a result of decreased recruitment or proliferation. We used Ki67 expression as a surrogate marker for proliferating cells and found a very high percentage of ILCs expressing Ki67, both non-activated (Lin^{-}ICOS^{+}CD45^{+}) and activated (cytokine secreting), but no significant difference in the frequency of proliferating total ILCs or ILC2s specifically in *Ccsp*-cre*Tgfb1*$^{-/-}$ mice (Figures 6D and 6E), indicating that TGF-β derived from the epithelium is essential for accumulation of ILCs in the airways.

Ccsp-cre*Tgfb1*$^{-/-}$ Mice Have Enhanced GATA-3 Regulation

GATA-3 is the master transcription factor for induction of Th2 cytokines in both T cells and ILCs (Hoyler et al., 2012). We assessed whether GATA-3 levels were reduced in *Ccsp*-cre*Tgfb1*$^{-/-}$ mice to account for their lower levels of Th2 cytokines such as IL-13 and IL-5. We found no change in GATA-3 expression in the lung either at the protein or mRNA level after TGF-β depletion in the pulmonary epithelium (Figures 6F–6H). However, control of GATA-3 activity is mediated via binding of regulatory elements to the transcription factor (rather than altered expression of GATA-3 protein), which enhance or retard binding to type 2 promoters and hence production of type 2 cytokines (Hossain et al., 2008; Kuwahara et al., 2012; Miaw et al., 2000; Zhou et al., 2001). We investigated the expression of known GATA-3 regulatory proteins and found no change in the mRNA levels of zinc finger protein multitype 1 (*Zfpm1*) (Figure 6I), Lymphoid Enhancer Factor 1 (*Lef1*) (Figure 6J), or zinc finger and BTB domain containing 32 (*Zbtb32*) (Figure 6K). In contrast, a significant increase in mRNA levels of the GATA-3-suppressive transcription factor *Sox4* (sex determining region Y, box 4) was observed in lung tissue of *Ccsp*-cre*Tgfb1*$^{-/-}$ mice (Figure 6L). In sorted cells, the increase in *Sox4* was found to be specific to ILCs and levels were unchanged in T cells from *Ccsp*-cre*Tgfb1*$^{-/-}$ mice (Figure 6M). Therefore, local TGF-β production can induce altered GATA-3 function via the increased expression of the GATA-3 regulatory protein Sox4.

TGF-β Acts to Enhance Airway ILC Chemoactivity

Next, we questioned whether the reduced number of ILCs in the airways was due to a deficiency in accumulation. TGF-β has previously been shown to increase cell migration of both immune and stromal cells via two mechanisms. It can act directly as a chemoattractant and indirectly by upregulating chemokine receptors and hence sensitivity to chemokines (Castriconi et al., 2013; Happel et al., 2008). With the TAXIScan chemotaxis assay system, we assessed the capacity of ILCs sorted from the airway lumen of rIL-33-treated mice to migrate along gradients of rIL-33 or rTGF-β (Figure 7A). We found that exposure of ILCs to a gradient of rTGF-β resulted in a significant increase in both the distance traveled by the ILCs (Figure 7B) and also their velocity (Figure 7C), when compared to cells exposed to PBS or rIL-33. However, this increased migration was non-directional, with no significant differences in the directionality component between either group (Figure 7D). We also examined the movement of ILCs from *Ccsp*-cre*Tgfb1*$^{-/-}$ mice, finding no significant difference in their migratory capacity compared to control mice (Figures S6I and S6J). Therefore, there were no intrinsic defects in the capacity of ILCs from *Ccsp*-cre*Tgfb1*$^{-/-}$ mice to respond to and be activated by pulmonary epithelial-derived TGF-β.

These data suggest that TGF-β does not act as a chemotactic factor for ILCs but rather increases the basal migration of ILCs, leading to greater overall cell movement. This might "prime" the cells for enhanced responsiveness to additional chemotactic stimuli. Taken together, our data demonstrate that the local

(B) FACs plot showing TGF-βRII expression on ILCs.
(C) TGF-βRII expression on ILCs from HDM and rIL-33-treated control mice.
(D and E) Proliferation defined as Ki67 expression on (D) ILC and (E) ILC2 (IL-13^{+}) populations in the lung of *Ccsp*-cre*Tgfb1*$^{-/-}$ (DOX) and *Tgfb1*$^{+/+}$ (MOCK) mice after rIL-33 (or PBS) administration.
(F and G) GATA-3 expression on (F) ILC and (G) ILC2 (IL-13^{+}) populations in the lung.
(H) mRNA levels of *Gata3* in the lung.
(I–L) mRNA levels of GATA-3 regulatory proteins (I) *Zfpm1*, (J) *Lef1*, (K) *Zbtb32*, and (L) *Sox4* in lung tissue.
(M) *Sox4* mRNA levels in FACs-sorted CD4^{+} T cells and ILCs.
Mann-Whitney *$p < 0.05$; n = 7–12 mice per group. Box and whisker plots depict the median and IQR and minimum and maximum values. Bar charts are expressed as mean ± SEM.

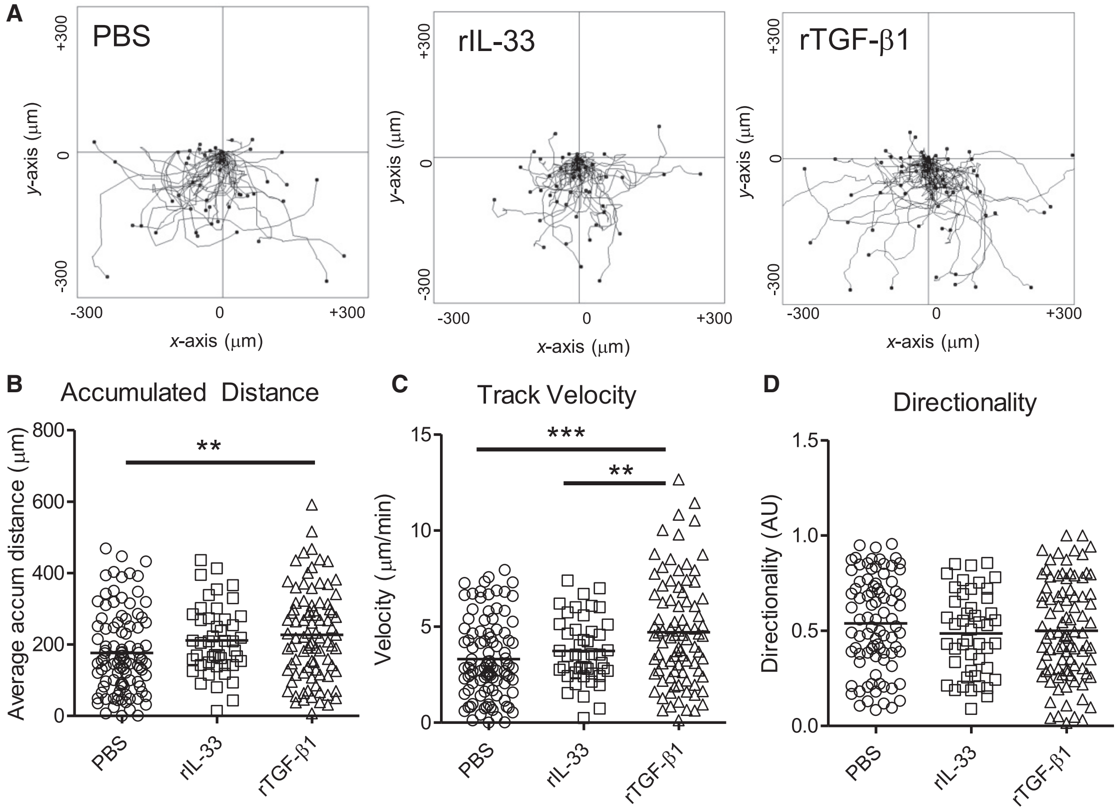

Figure 7. TGF-β Induced Chemoactivity

(A) Plots showing cell tracking of individual migrating cells exposed to chemoattractant gradients over 60 min. Cells were realigned to show the same point of origin.

(B–D) Accumulated distance (B), track velocity (C), and directionality (D) of individual cells. ILCs (lineage^{-}CD45^{+}ICOS^{+}) were recovered from the airway lumen of control (*Tgfb1*$^{+/+}$) mice treated with rIL-33 for 1 week. Cells were then exposed to gradients of rIL-33 (20 μg/ml), rTGF-β1 (5 μg/ml), or PBS and cell movement assessed by TAXIScan methodology.

Data were pooled from three individual experiments with a minimum total of $n \geq 50$ cells tracked per group. Dot plots depict median values. See also Figure S6.

mucosal environment and specifically epithelial-derived TGF-β is a crucial driver of the early allergic immune response to inhaled stimuli in the lung.

DISCUSSION

Pulmonary epithelial cells play a pivotal role in both the maintenance of immune homeostasis and defense against pathogens in the lung. In order to investigate the cellular networks required for the molecular pathways that maintain this balance between tolerance to inhaled particles and development of inflammation, we generated inducible bronchial epithelial cell-specific knockouts of TGF-β1. In contrast to global and T cell-specific TGF-β knockouts, mice lacking epithelial-derived TGF-β displayed no baseline immune defects but were protected from pathophysiological consequences upon exposure to inhaled allergen. In response to HDM exposure, *Ccsp*-cre*Tgfb1*$^{-/-}$ mice displayed a reduction in key features of asthma pathology, namely diminished AHR, eosinophilia, pulmonary inflammation, type 2 cytokines, and IL-4-driven IgE production. We have determined that this lack of IL-13-driven allergic pathophysiology was specifically due to a reduction in pulmonary IL-13^{+} ILC2 activation and accumulation.

Moreover, our data show that ILCs in the airway lumen are primed to respond to TGF-β, as a result of high cell surface expression of TGF-βRII, and we demonstrated that TGF-β enhanced the migratory activity of ILCs. Delivery of rIL-33 to the lung, which is known to result in the generation of ILC2s, induced a rapid release of TGF-β into the airways. Because *Ccsp*-cre*Tgfb1*$^{-/-}$ mice had limited numbers of pulmonary ILCs, one can infer that epithelial-derived TGF-β is essential for the accumulation of these cells in the lung, acting downstream of the alarmin IL-33 to initiate a type 2 skewed immune response. In summary, this interaction between ILCs and TGF-β derived from the lung epithelium is a key pathway in the generation of allergic phenotype.

A variety of resident pulmonary cells are able to secret TGF-β, including lung stromal cells as well as infiltrating leukocytes. Here we showed that specific depletion of TGF-β in the bronchial epithelium (rather than alveolar epithelial cells or alveolar macrophages) resulted in a very selective immune defect with profound consequences for disease pathology. Epithelial cells in the lung act as sensors, sampling the inhaled environment. The activation of pathogen-associated molecular pattern molecules (PAMPs) and damage-associated molecular pattern molecules (DAMPs), including the alarmin IL-33 in epithelial cells, both initiates and

perpetuates the inflammatory response to pathogens and allergens. Thus, local environmental cues influence both cytokine production and receptor expression on structural cells and leukocytes. Our findings place TGF-β as a potent modulator of local immune responses with substantial consequences for systemic immunity.

We demonstrated that a greater proportion of ILCs expressed TGF-βRII in the airway lumen compared to any other compartment examined. These data indicated that IL-13$^+$ ILC2s might be intimately associated with the pulmonary epithelium and that their function is influenced by the local cytokine milieu. Indeed, a previous study has shown that the local cytokine environment is critical for the generation of distinct cytokine signatures. A unique human ILC-1 subset was identified that showed the hallmark of TGF-β imprinting and activation (Fuchs et al., 2013). Interestingly, this subset was shown to reside specifically in the intraepithelial niche within the gut. Although the authors did not comment on the in vivo cellular source for TGF-β in the gut, it is known that intestinal epithelial cells, as in the lung, can express TGF-β (Avery et al., 1993). Accumulation of cells within tissue is the net result of recruitment and/or proliferation. We determined that TGF-β enhances the migratory capacity of ILCs, which are highly proliferative, collectively resulting in increased local numbers of these cells. The pulmonary epithelium expresses a range of molecules that enable rapid sensing and reaction to inhaled particles (Lambrecht and Hammad, 2012). The data presented here add TGF-β to this list and support the hypothesis that interaction between local tissue-resident cells and leukocytes dictates the development of either protective or inflammatory immune responses.

GATA-3 is a requirement for generation of the ILC2 phenotype, with ectopic expression of GATA-3 inducing de novo expression of type 2 cytokines (Hoyler et al., 2012; Zhou, 2012). We hypothesized that the reduction in type 2 cytokines in *Ccsp-creTgfb1*$^{-/-}$ mice was due to a change in GATA-3 expression; however, we found no change in the proportion of GATA-3-positive ILCs. However, we determined that specific deletion of TGF-β from the cells lining the airways was associated with increased levels of the negative GATA-3 regulator Sox4 during allergen exposure. It has previously been shown that Sox4 transgenic mice exhibit decreased ovalbumin-induced allergic airway disease with lower AHR, IL-5, and eosinophilia (Kuwahara et al., 2012). In contrast, Sox4 knockout mice show the opposite phenotype and develop enhanced allergic immune responses. Our data revealed that epithelial-specific deletion of TGF-β resulted in increased expression of *Sox4* in ILCs that inhibited GATA-3-induced type 2 cytokine expression. Therefore, location of cytokine-secreting cells is very important in determining the nature, magnitude, and bias of the subsequent immune response.

ILCs are considered to be generated from an Id2$^+$ precursor population resident in the bone marrow that proliferate and traffic though the blood to mucosal sites. Very little is understood about the process by which ILCs are attracted to sites of immune insult. Other innate and adaptive lymphoid populations express a distinct pattern of chemokine and cytokine receptors and are attracted to tissues via chemokine gradients. Recently, both PGD_2 and to a much lesser extent IL-33 have been shown to be chemotactic for human skin- and blood-derived ILC2s (Xue et al., 2014). However, as yet, we lack a complete understanding of the repertoire of chemotactic factors and agents utilized by ILCs. We have shown that TGF-β acts as a chemoactive factor for ILCs. Previously, TGF-β has been shown to be required both for the migration and maintenance of memory T cell populations at the gut mucosa (Zhang and Bevan, 2013). In combination, these findings underscore the critical role of TGF-β in controlling the recruitment and retention of specific cell populations at mucosal surfaces.

Our data highlight the crucial role that TGF-β plays in regulating immune responses, particularly at mucosal surfaces where the threshold for generating immune responses must be exquisitely regulated. Evidence for an association between the innate cytokine IL-33 and TGF-β can be found in the gut where IL-33 has recently been shown to enhance TGF-β1-mediated differentiation of Treg cells in the bowel (Schiering et al., 2014). In the lung, we have unraveled a pathway for initiation of Th2 cell responses whereby IL-33 directly induces TGF-β release from the pulmonary epithelium. IL-33-induced TGF-β enhances migration of ILCs, which express the TGF-βRII. Thus, TGF-β is critical for the development of a robust ILC2 response to allergen challenge and initiation of allergic immunity. Our data provide direct evidence for the instruction of immune cells by resident stromal cells and reinforce the idea that the local environmental milieu is pivotal in defining the nature and magnitude of immune reactions.

EXPERIMENTAL PROCEDURES

Animals and Reagents

Mice expressing Cre recombinase under the control of the rat CCSP promoter *CCSP*-$_{rtTA/tetO}$-Cre (Perl et al., 2009) were crossed with mice carrying floxed alleles for *Tgfb1* (Azhar et al., 2009) (Ja*Tgfb1*$^{tm2.1Doe}$/J, Jackson Laboratory). Intraperitoneal administration of 2 mg doxycycline (DOX) (Sigma-Aldrich) induced epithelial expression of Cre and excision of TGF-β1 (*Ccsp-creTgfb1*$^{-/-}$). Control mice included mock (100 μl PBS i.p.)-treated mice that received no DOX (*Tgfb1*$^{+/+}$) and littermate controls treated with DOX or mock, which carried homozygous floxed *Tgfb1* but lacked either the *CCSP*-$_{rtTA}$ (or $_{tetO}$-Cre) allele and were therefore unable to excise *Tgfb1*. All mice were used between 7 and 12 weeks of age, housed in specific-pathogen-free conditions, and given food and water ad libitum. All procedures were conducted in accordance with the Animals (Scientific Procedures) Act 1986.

Mice were administered either 25 μg (1 mg/ml protein weight solution dissolved in PBS) of house dust mite (HDM) extract (Greer) or 25 μl of PBS intranasally 5 times a week for 3 weeks. Mice were culled 4 hr after the final HDM or PBS dose. Carrier-free recombinant murine IL-33 (1 μg per dose in 25 μl PBS) (eBioscience) was administered 3 times a week for 1 week and mice culled 18 hr after the final dose. In other experiments, mice were administered 50 ng rTGF-β1 in PBS (R&D Systems) intranasally with or without rIL-33.

Lung Function

Airway hyperresponsiveness in response to increasing doses of methacholine (10–300 mg/ml; Sigma-Aldrich) was measured as previously described using the Flexivent system (Scireq) (Saglani et al., 2009).

Cell Recovery

Bronchoalveolar lavage (BAL) was performed by washing the airways three times with 400 μl of PBS, after centrifugation supernatants were stored at −80°C for further analysis and cells resuspended in 500 μl of complete media (RPMI, 10% FCS, 2 mM L-glutamine, 100 U/ml penicillin/streptomycin) (GIBCO, Life Technologies). The left and inferior lung lobes were chopped and digested in complete media supplemented with 0.15 mg/ml collagenase (Type D; Roche Diagnostics) and 25 μg/ml DNase (Type 1; Roche Diagnostics) for 1 hr at 37°C. Tissue was then passed through a 70 μm sieve (BD

Bioscience), washed, and resuspended in 1 ml of complete media. Bone marrow (recovered from the left femur) and mediastinal lung draining lymph nodes were passed through a 70 μm sieve, washed, and resuspended in 1 ml of complete media. Red blood cells in 200 μl whole blood were lysed and the remaining leukocytes were washed twice and then resuspended in 1 ml of complete media.

Flow Cytometry

For intracellular staining of cytokines, cells were stimulated with PMA (Sigma-Aldrich)/ionomycin (Emdchemicals) in the presence of Brefeldin A (Sigma-Aldrich) for 3.5 hr at 37°C. Cells were washed and incubated for 20 min with rabbit serum (Sigma-Aldrich) prior to staining for extracellular antigens in 5% FCS/1% BSA in PBS for 30 min at 4°C. All antibodies were purchased from eBioscience with the exception of ICOS (Biolegend), TGF-βRII (R&D Systems), and T1/ST2 (MD Bioproducts). Cells were washed, fixed, and permeabilized using Fix/Perm kit (eBioscience) before being stained for intracellular antigens. Analysis was performed with Fortessa II and cell sorting on Aria III (BD Biosciences).

Club/Clara Cell Sorting

Club/Clara cells were sorted as described (Wang et al., 2012) prior to determining the level of *Tgfb1* mRNA in the club ($CCSP^{+}CD45^{-}$) and $CCSP^{-}CD45^{+}$ cell fractions.

Measurement of Cytokines and Immunoglobulins

The middle lung lobe was homogenized at 50 mg/ml in HBSS (GIBCO) containing protease inhibitor tablets (Roche Diagnostics) and centrifuged at 800 × *g* for 20 min. Lung homogenate supernatants were used to analyze cytokines and immunoglobulins and serum to analyze immunoglobulins. Paired antibodies for murine IgE, IgA, IL-4, IL-5 (BD Biosciences), IL-13 (eBioscience), IL-33, KC, MIP-2, Eotaxin-2, and MCP-1 (R&D Systems) were used in standardized sandwich ELISAs according to the manufacturer's protocol. HDM-specific IgE was measured in the serum as previously described (Saglani et al., 2009). TGF-β in BAL fluid was measured by bioassay as previously described (Tesseur et al., 2006).

Lung Inflammation Scoring

Haematoxylin and eosin-stained lung sections were scored blindly for lung infiltrates where scores correspond to 1 (small pocket of infiltrate), 2 (small pocket of infiltrate less than three cells deep in more than one airway or vessel), 3 (more than one but less than 50% of airways and vessels have large infiltrates more than three cells deep), 4 (most airways and vessels have large infiltrates), and 5 (majority of airways and vessels have large infiltrates and cells present in alveolar bed).

Real-Time PCR

Total RNA was extracted from 50 to 100 mg of lung tissue (postcaval lobe) using a QIAGEN RNeasy Mini Kit (QIAGEN). Total RNA (1 μg) was reverse transcribed into cDNA using a High Capacity cDNA Reverse Transcription Kit (Life Technologies) according to the manufacturer's instructions. Real-time PCR reactions were performed using fast-qPCR mastermix (Life Technologies) on a Viaa-7 instrument (Life Technologies) with TaqMan primer sets for murine *Il13*, *Il5*, *Gata-3*, *Zfpm1*, *Lef1*, *Zbtb32*, *Sox4*, *Tgfb1*, *Tgfb2*, and *Tgfb3* plus housekeeping genes *Gapdh* and *Hprt* (Life Technologies). Gene expression was analyzed using the change-in-threshold ΔΔCt- method using *Hprt* as control.

Immunofluorescence and Immunohistochemistry

Paraffin sections were stained with rabbit anti-cre antibody (Merck Millipore) or rabbit anti-TGF-β antibody (BD Biosciences) using an avidin/biotin staining method.

TAXIScan Chemotaxis Assay

Control (*Tgfb1*$^{+/+}$) and *Ccsp*-cre*Tgfb1*$^{-/-}$ mice were administered rIL-33 and cells recovered from the airway lumen. ILC population ($lineage^{-}CD45^{+}ICOS^{+}$) was sorted using an Aria III (BD Biosciences). For real-time analysis of migrating ILCs, a 12-channel TAXIScan was used in conjunction with a 4 μm chip as previously described (Kanegasaki et al., 2003), according to the manufacturer's protocol (Effector Cell Institute). After alignment of cells, a 1 μl volume of rIL-33 (20 μg/ml), rTGF-β (5 μg/ml), or PBS (negative control) was then added to the side of the chamber opposite the cells to generate a gradient.

Sequential images of migrating cells were generated from individual jpegs processed with ImageJ (NIH, RRID: nif-0000-30467), equipped with the manual tracking and chemotaxis tool plugins (Ibidi). Directionality was calculated by dividing the Euclidean distance by the observed accumulated distance for each tracked cell, with a value of 1 indicative of linear migration. Accumulated distances refer to the total distances traveled by a particular cell.

Statistical Analysis

All data were analyzed with Graph Pad Prism 5 (GraphPad, RRID: rid_000081). Box and whisker plots depict the median and IQR and line graphs and bar charts are expressed as mean ± SEM and data analyzed with non-parametric Mann-Whitney U test where significance was defined as $^{*}p < 0.05$, $^{**}p < 0.01$, and $^{***}p < 0.001$.

SUPPLEMENTAL INFORMATION

Supplemental Information includes six figures and can be found with this article online at http://dx.doi.org/10.1016/j.immuni.2015.10.012.

AUTHOR CONTRIBUTIONS

C.M.L. conceived the idea and directed the study. L.D., L.G.G., and C.M.L. wrote the manuscript. L.D. and C.M.L. designed the experiments and L.D., L.G.G., T.J.S., J.S.B., J.E.P., G.M.F.H., and S.A.W. performed experiments. L.D., L.G.G., T.J.S., J.S.B., S.A.W., and A.J.B. analyzed the data and L.D., L.G.G., and A.J.B. generated figures.

ACKNOWLEDGMENTS

The authors thank Catherine Simpson and Jane Srivastava of the Imperial College Core Flow Cytometry facility for assistance with cell sorting, Lorraine Lawrence for histological sectioning, and staff of the Mary Lyon Centre (MLC) at Harwell for performing mouse re-derivation and animal husbandry support. We are grateful to Shiro Kanegasaki and Tomoko Tsuchiya of the YU-ECI Research Center for Medical Science, Yeungnam University, Republic of Korea, for their help and support with the use of the 12-channel TAXIScan system. This work was supported by the Wellcome Trust (grant 087618/Z/08/Z).

Received: November 27, 2014
Revised: June 16, 2015
Accepted: August 5, 2015
Published: November 17, 2015

REFERENCES

Avery, A., Paraskeva, C., Hall, P., Flanders, K.C., Sporn, M., and Moorghen, M. (1993). TGF-beta expression in the human colon: differential immunostaining along crypt epithelium. Br. J. Cancer *68*, 137–139.

Azhar, M., Yin, M., Bommireddy, R., Duffy, J.J., Yang, J., Pawlowski, S.A., Boivin, G.P., Engle, S.J., Sanford, L.P., Grisham, C., et al. (2009). Generation of mice with a conditional allele for transforming growth factor beta 1 gene. Genesis *47*, 423–431.

Barlow, J.L., Peel, S., Fox, J., Panova, V., Hardman, C.S., Camelo, A., Bucks, C., Wu, X., Kane, C.M., Neill, D.R., et al. (2013). IL-33 is more potent than IL-25 in provoking IL-13-producing nuocytes (type 2 innate lymphoid cells) and airway contraction. J. Allergy Clin. Immunol. *132*, 933–941.

Castriconi, R., Dondero, A., Bellora, F., Moretta, L., Castellano, A., Locatelli, F., Corrias, M.V., Moretta, A., and Bottino, C. (2013). Neuroblastoma-derived TGF-β1 modulates the chemokine receptor repertoire of human resting NK cells. J. Immunol. *190*, 5321–5328.

Chen, W., Jin, W., Hardegen, N., Lei, K.J., Li, L., Marinos, N., McGrady, G., and Wahl, S.M. (2003). Conversion of peripheral CD4+CD25- naive T cells to CD4+CD25+ regulatory T cells by TGF-beta induction of transcription factor Foxp3. J. Exp. Med. *198*, 1875–1886.

Dardalhon, V., Awasthi, A., Kwon, H., Galileos, G., Gao, W., Sobel, R.A., Mitsdoerffer, M., Strom, T.B., Elyaman, W., Ho, I.C., et al. (2008). IL-4 inhibits TGF-beta-induced Foxp3+ T cells and, together with TGF-beta, generates IL-9+ IL-10+ Foxp3(-) effector T cells. Nat. Immunol. *9*, 1347–1355.

de Boer, W.I., van Schadewijk, A., Sont, J.K., Sharma, H.S., Stolk, J., Hiemstra, P.S., and van Krieken, J.H. (1998). Transforming growth factor beta1 and recruitment of macrophages and mast cells in airways in chronic obstructive pulmonary disease. Am. J. Respir. Crit. Care Med. *158*, 1951–1957.

Fattouh, R., Midence, N.G., Arias, K., Johnson, J.R., Walker, T.D., Goncharova, S., Souza, K.P., Gregory, R.C., Jr., Lonning, S., Gauldie, J., and Jordana, M. (2008). Transforming growth factor-beta regulates house dust mite-induced allergic airway inflammation but not airway remodeling. Am. J. Respir. Crit. Care Med. *177*, 593–603.

Fuchs, A., Vermi, W., Lee, J.S., Lonardi, S., Gilfillan, S., Newberry, R.D., Cella, M., and Colonna, M. (2013). Intraepithelial type 1 innate lymphoid cells are a unique subset of IL-12- and IL-15-responsive IFN-γ-producing cells. Immunity *38*, 769–781.

Gibbs, J.D., Ornoff, D.M., Igo, H.A., Zeng, J.Y., and Imani, F. (2009). Cell cycle arrest by transforming growth factor beta1 enhances replication of respiratory syncytial virus in lung epithelial cells. J. Virol. *83*, 12424–12431.

Gregory, L.G., Mathie, S.A., Walker, S.A., Pegorier, S., Jones, C.P., and Lloyd, C.M. (2010). Overexpression of Smad2 drives house dust mite-mediated airway remodeling and airway hyperresponsiveness via activin and IL-25. Am. J. Respir. Crit. Care Med. *182*, 143–154.

Gregory, L.G., Jones, C.P., Mathie, S.A., Pegorier, S., and Lloyd, C.M. (2013). Endothelin-1 directs airway remodeling and hyper-reactivity in a murine asthma model. Allergy *68*, 1579–1588.

Happel, C., Steele, A.D., Finley, M.J., Kutzler, M.A., and Rogers, T.J. (2008). DAMGO-induced expression of chemokines and chemokine receptors: the role of TGF-beta1. J. Leukoc. Biol. *83*, 956–963.

Hossain, M.B., Hosokawa, H., Hasegawa, A., Watarai, H., Taniguchi, M., Yamashita, M., and Nakayama, T. (2008). Lymphoid enhancer factor interacts with GATA-3 and controls its function in T helper type 2 cells. Immunology *125*, 377–386.

Hoyler, T., Klose, C.S., Souabni, A., Turqueti-Neves, A., Pfeifer, D., Rawlins, E.L., Voehringer, D., Busslinger, M., and Diefenbach, A. (2012). The transcription factor GATA-3 controls cell fate and maintenance of type 2 innate lymphoid cells. Immunity *37*, 634–648.

Jones, C.P., Gregory, L.G., Causton, B., Campbell, G.A., and Lloyd, C.M. (2012). Activin A and TGF-β promote T(H)9 cell-mediated pulmonary allergic pathology. J. Allergy Clin. Immunol. *129*, 1000–10.e3.

Kanegasaki, S., Nomura, Y., Nitta, N., Akiyama, S., Tamatani, T., Goshoh, Y., Yoshida, T., Sato, T., and Kikuchi, Y. (2003). A novel optical assay system for the quantitative measurement of chemotaxis. J. Immunol. Methods *282*, 1–11.

Kariyawasam, H.H., Pegorier, S., Barkans, J., Xanthou, G., Aizen, M., Ying, S., Kay, A.B., Lloyd, C.M., and Robinson, D.S. (2009). Activin and transforming growth factor-beta signaling pathways are activated after allergen challenge in mild asthma. J. Allergy Clin. Immunol. *124*, 454–462.

Kearley, J., Barker, J.E., Robinson, D.S., and Lloyd, C.M. (2005). Resolution of airway inflammation and hyperreactivity after in vivo transfer of CD4+CD25+ regulatory T cells is interleukin 10 dependent. J. Exp. Med. *202*, 1539–1547.

Kim, H.Y., Chang, Y.J., Subramanian, S., Lee, H.H., Albacker, L.A., Matangkasombut, P., Savage, P.B., McKenzie, A.N., Smith, D.E., Rottman, J.B., et al. (2012). Innate lymphoid cells responding to IL-33 mediate airway hyperreactivity independently of adaptive immunity. J. Allergy Clin. Immunol. *129*, 216–27.e1, 6.

Kulkarni, A.B., Huh, C.G., Becker, D., Geiser, A., Lyght, M., Flanders, K.C., Roberts, A.B., Sporn, M.B., Ward, J.M., and Karlsson, S. (1993). Transforming growth factor beta 1 null mutation in mice causes excessive inflammatory response and early death. Proc. Natl. Acad. Sci. USA *90*, 770–774.

Kuwahara, M., Yamashita, M., Shinoda, K., Tofukuji, S., Onodera, A., Shinnakasu, R., Motohashi, S., Hosokawa, H., Tumes, D., Iwamura, C., et al. (2012). The transcription factor Sox4 is a downstream target of signaling by the cytokine TGF-β and suppresses T(H)2 differentiation. Nat. Immunol. *13*, 778–786.

Lambrecht, B.N., and Hammad, H. (2012). The airway epithelium in asthma. Nat. Med. *18*, 684–692.

Li, M.O., and Flavell, R.A. (2008). TGF-beta: a master of all T cell trades. Cell *134*, 392–404.

Li, M.O., Wan, Y.Y., Sanjabi, S., Robertson, A.K., and Flavell, R.A. (2006). Transforming growth factor-beta regulation of immune responses. Annu. Rev. Immunol. *24*, 99–146.

Li, H., Romieu, I., Wu, H., Sienra-Monge, J.J., Ramírez-Aguilar, M., del Río-Navarro, B.E., del Lara-Sánchez, I.C., Kistner, E.O., Gjessing, H.K., and London, S.J. (2007). Genetic polymorphisms in transforming growth factor beta-1 (TGFB1) and childhood asthma and atopy. Hum. Genet. *121*, 529–538.

Licona-Limón, P., Kim, L.K., Palm, N.W., and Flavell, R.A. (2013). TH2, allergy and group 2 innate lymphoid cells. Nat. Immunol. *14*, 536–542.

McMillan, S.J., Xanthou, G., and Lloyd, C.M. (2005). Manipulation of allergen-induced airway remodeling by treatment with anti-TGF-beta antibody: effect on the Smad signaling pathway. J. Immunol. *174*, 5774–5780.

Miaw, S.C., Choi, A., Yu, E., Kishikawa, H., and Ho, I.C. (2000). ROG, repressor of GATA, regulates the expression of cytokine genes. Immunity *12*, 323–333.

Perl, A.K., Zhang, L., and Whitsett, J.A. (2009). Conditional expression of genes in the respiratory epithelium in transgenic mice: cautionary notes and toward building a better mouse trap. Am. J. Respir. Cell Mol. Biol. *40*, 1–3.

Saglani, S., Mathie, S.A., Gregory, L.G., Bell, M.J., Bush, A., and Lloyd, C.M. (2009). Pathophysiological features of asthma develop in parallel in house dust mite-exposed neonatal mice. Am. J. Respir. Cell Mol. Biol. *41*, 281–289.

Schiering, C., Krausgruber, T., Chomka, A., Fröhlich, A., Adelmann, K., Wohlfert, E.A., Pott, J., Griseri, T., Bollrath, J., Hegazy, A.N., et al. (2014). The alarmin IL-33 promotes regulatory T-cell function in the intestine. Nature *513*, 564–568.

Schultz-Cherry, S., and Hinshaw, V.S. (1996). Influenza virus neuraminidase activates latent transforming growth factor beta. J. Virol. *70*, 8624–8629.

Shull, M.M., Ormsby, I., Kier, A.B., Pawlowski, S., Diebold, R.J., Yin, M., Allen, R., Sidman, C., Proetzel, G., Calvin, D., et al. (1992). Targeted disruption of the mouse transforming growth factor-beta 1 gene results in multifocal inflammatory disease. Nature *359*, 693–699.

Silverman, E.S., Palmer, L.J., Subramaniam, V., Hallock, A., Mathew, S., Vallone, J., Faffe, D.S., Shikanai, T., Raby, B.A., Weiss, S.T., and Shore, S.A. (2004). Transforming growth factor-beta1 promoter polymorphism C-509T is associated with asthma. Am. J. Respir. Crit. Care Med. *169*, 214–219.

Sullivan, D.E., Ferris, M., Nguyen, H., Abboud, E., and Brody, A.R. (2009). TNF-alpha induces TGF-beta1 expression in lung fibroblasts at the transcriptional level via AP-1 activation. J. Cell. Mol. Med. *13* (8B), 1866–1876.

Tesseur, I., Zou, K., Berber, E., Zhang, H., and Wyss-Coray, T. (2006). Highly sensitive and specific bioassay for measuring bioactive TGF-beta. BMC Cell Biol. *7*, 15.

Travis, M.A., and Sheppard, D. (2014). TGF-β activation and function in immunity. Annu. Rev. Immunol. *32*, 51–82.

van Ginkel, F.W., Wahl, S.M., Kearney, J.F., Kweon, M.N., Fujihashi, K., Burrows, P.D., Kiyono, H., and McGhee, J.R. (1999). Partial IgA-deficiency with increased Th2-type cytokines in TGF-beta 1 knockout mice. J. Immunol. *163*, 1951–1957.

Van Stry, M., and Bix, M. (2011). Explaining discordant coordination. Nat. Immunol. *12*, 16–17.

Vareille, M., Kieninger, E., Edwards, M.R., and Regamey, N. (2011). The airway epithelium: soldier in the fight against respiratory viruses. Clin. Microbiol. Rev. *24*, 210–229.

Walker, J.A., and McKenzie, A. (2012). Innate lymphoid cells in the airways. Eur. J. Immunol. *42*, 1368–1374.

Wang, X.Y., Keefe, K.M., Jensen-Taubman, S.M., Yang, D., Yan, K., and Linnoila, R.I. (2012). Novel method for isolation of murine clara cell secretory protein-expressing cells with traces of stemness. PLoS ONE *7*, e43008.

Winkelmann, A., and Noack, T. (2010). The Clara cell: a ''Third Reich eponym''? Eur. Respir. J. *36*, 722–727.

Xue, L., Salimi, M., Panse, I., Mjösberg, J.M., McKenzie, A.N., Spits, H., Klenerman, P., and Ogg, G. (2014). Prostaglandin D2 activates group 2 innate lymphoid cells through chemoattractant receptor-homologous molecule expressed on TH2 cells. J. Allergy Clin. Immunol. *133*, 1184–1194.

Zhang, N., and Bevan, M.J. (2013). Transforming growth factor-β signaling controls the formation and maintenance of gut-resident memory T cells by regulating migration and retention. Immunity *39*, 687–696.

Zhou, L. (2012). Striking similarity: GATA-3 regulates ILC2 and Th2 cells. Immunity *37*, 589–591.

Zhou, M., Ouyang, W., Gong, Q., Katz, S.G., White, J.M., Orkin, S.H., and Murphy, K.M. (2001). Friend of GATA-1 represses GATA-3-dependent activity in CD4+ T cells. J. Exp. Med. *194*, 1461–1471.

Immunity

Supplemental Information

Pulmonary Epithelial Cell-Derived Cytokine TGF-β1 Is a Critical Cofactor for Enhanced Innate Lymphoid Cell Function

Laura Denney, Adam J. Byrne, Thomas J. Shea, James S. Buckley, James E. Pease, Gaelle M.F. Herledan, Simone A. Walker, Lisa G. Gregory, and Clare M. Lloyd

Figure S1, related to Figure 1. Schematic of the generation of epithelial TGF-β knockout mice and the subsequent expression of Cre and TGF-β1 in the lung. (A) Double transgenic mice *CCSP*-$_{rtTA/tetO}$-Cre are crossed with mice expressing homozygous *Tgfb1* genes with LoxP sites (Floxed) and treated with doxycycline (DOX) at 6weeks of age. Upon DOX administration the Tet-on system is activated and cells expressing club cell secretory protein (CCSP) transcribe Cre recombinase which binds LoxP sites and excises exon 6 of the *Tgfb1* gene leading to the transcription of truncated and non-functional TGF-β. Littermate control mice lack one transgenic allele and are without a functional Tet-on system so therefore after DOX treatment continue to express TGF-β. (B) Immunohistochemistry images showing Cre expression 48hr post DOX (or mock) treatment of transgenic mice and littermate controls. (C) Immunohistochemistry images showing TGF-β expression 72hr post Dox treatment of transgenic mice and littermate controls. (D) mRNA levels of *Tgfb1* in sorted club cells ($CCSP^{+}CD45^{neg}$) and $CCSP^{neg}CD45^{+}$ cells in control and *Ccsp*-cre*Tgfb1*$^{-/-}$ mice. n=3 groups of >5 mice per group. Mann–Whitney *P<0.05. (E-H) Cell counts in the (E) blood, (F) spleen, (G) bone marrow and (H) mediastenial lymph nodes in *Ccsp*-cre*Tgfb1*$^{-/-}$ mice (DOX) and *Tgfb1*$^{+/+}$ (mock) treated mice 7, 21 and 42 days after treatment. Box and whisker plots depict the median and IQR and minimum and maximum values. Bar charts are expressed as mean +/- SEM. n=4-5 mice per group (Mann–Whitney).

Figure S2, related to Figure 1. Doxycycline administration alone does not result in an altered allergic lung phenotype (A) *Tgfb1*, *Tgfb2*, and *Tgfb3* mRNA levels in whole lung tissue of *Ccsp*-cre*Tgfb1*$^{-/-}$ mice (DOX) and *Tgfb1*$^{+/+}$ (mock) treated mice after HDM administration. Representative data is shown from 2 independent experiments with a total of n=8-12 mice per group (Mann–Whitney). (B-C) Airway hyperreactivity after intranasal house dust mite (HDM) or PBS administration as measured by (B) resistance and (C) compliance to an ascending methacholine concentration challenge in littermate control mice lacking either *CCSP*-$_{\text{rtTA}}$ or $_{\text{tetO}}$-Cre unable (upon DOX administration) to ablate TGF–β expression in their epithelium (baseline; BL). (D-F) Cell counts in the (D) BAL and (E) lung tissue after Dox treatment and HDM exposure and (F) Numbers of macrophages (MAC), eosinophils (EOS) and neutrophils (NEU) in the airways of littermate control mice lacking either *CCSP*-$_{\text{rtTA}}$ or $_{\text{tetO-}}$Cre. (G) Airway hyperreactivity measured by airway compliance to ascending methacholine concentration in *Ccsp*-cre*Tgfb1*$^{-/-}$ mice (DOX) and *Tgfb1*$^{+/+}$ (mock) treated mice (baseline; BL) (H) Histological scoring of lung inflammation after HDM (or PBS) treatment. (I) Eosinophils, macrophages and neutrophils in the lung tissue. Box and whisker plots depict the median and IQR and minimum and maximum values. Line graphs and bar graphs are expressed as mean +/- SEM. n=4-6 mice per group (Mann–Whitney).

Figure S3, related to Figures 2 and 3. Phenotype of *Ccsp*-cre*Tgfb1*$^{-/-}$ mice treated with HDM. (A-C) Levels of (A) KC, (B) MIP-2 and (C) MCP-1 in the lung tissue. (D) IgA levels in the BAL. (E) Flow cytometry plots show Th2 (CD3^{+}CD4^{+}IL-13^{+}) cell gating strategy example shown is HDM treated mouse lung. (F-G) Frequencies of pulmonary T cells (F) IL-4^{+} T cells in the lung and (G) Th17 cells and (H) Th1 cells in the BAL. (I-J) Levels of (I) IL-17A and (J) IFN-γ levels in the lung. Representative data is shown from 2 independent experiments, with a total of n=8-12 mice per group, Mann–Whitney *P<0.05. Box and whisker plots depict the median and IQR and minimum and maximum values.

Figure S4, related to Figure 3. Innate lymphoid cell identification and gating. (A) Flow cytometry gating strategy for IL-13^{+} Lin^{neg}ICOS^{+}CD45^{+} ILCs where lineage cocktail contains CD3, CD45R, CD11b, TER-119, Ly-6G (GR1). (B) Flow cytometry gating strategy for IL-13^{+}, IL-4^{+} and IL-5^{+} Lin^{neg}ICOS^{+}CD45^{+} ILCs. (C) Flow cytometry plots depicting Lin^{neg} and Lin^{+} populations with additional linage markers (CD5, CD4, NK1.1, CD8, CD11c, β-TCR, $\gamma\delta$-TCR. (D) Plots show expression of ST2, CD127 and Thy1 on IL-13^{+} Lin^{neg}ICOS^{+}CD45^{+} ILC population.

Figure S5, related to Figures 3 and 4. Innate cells in the lung (A) Flow cytometry plots depicting IL-5^{+} and IL-13^{+} ILCs from PBS treated control mice and HDM treated *Ccsp*-cre*Tgfb1*$^{-/-}$ mice and littermate controls. (B) IL-4^{+} ILC numbers in the lung. (C) IL-5^{+} ILC numbers in the lung. (D-E) GATA-3^{+} ILCs in the (D) lung and (E) BAL. (F) IL-33 levels in the lung. (G) NK cell (NKp46^{+} CD3^{neg}) numbers in lung tissue of *Ccsp*-cre*Tgfb1*$^{-/-}$ mice (DOX) and *Tgfb1*$^{+/+}$ (mock) treated mice. (H) Frequency of ILC3 defined as IL-17A^{+} lineage^{neg}CD45^{+}ICOS^{+} in lung. (I) Histogram showing T1/ST2 expression on club cells (CCSP^{+}CD45^{neg}) recovered from the lung of naïve mice. (J) Levels of bioactive TGF-β in the BAL of control and *Ccsp*-cre*Tgfb1*$^{-/-}$ mice. Representative data is shown from 2 independent experiments, with a total of n=8-12 mice per group Mann–Whitney *P<0.05 and **P<0.01 Box and whisker plots depict the median and IQR and minimum and maximum values.

Figure S6, related to Figures 4 and 7. Phenotype of *Ccsp*-cre*Tgfb1*$^{-/-}$ mice mice treated with rIL-33. (A) Eosinophils (EOS), macrophages (MAC) and neutrophils (NEU) in the lung tissue. (B) Levels of IL-5 and (C) eotaxin-2 in the lung. (D) Total cell counts and (E) eosinophil numbers in the BAL. (F) Th2 cells and (G) ILC2 numbers in the BAL. (H) IL-13 levels in the BAL. (I) Accumulated distance and (J) track velocity of ILCs from control (*Tgfb1*$^{+/+}$) and *Ccsp*-cre*Tgfb1*$^{-/-}$ mice exposed to gradients of rTGF-β1 (5μg/ml) assessed using TAXIScan methodology. Representative data is shown from 2 independent experiments, with a total of n=8-12 mice per group. Box and whisker plots depict the median and IQR and minimum and maximum values. Bar graphs are expressed as mean +/- SEM.

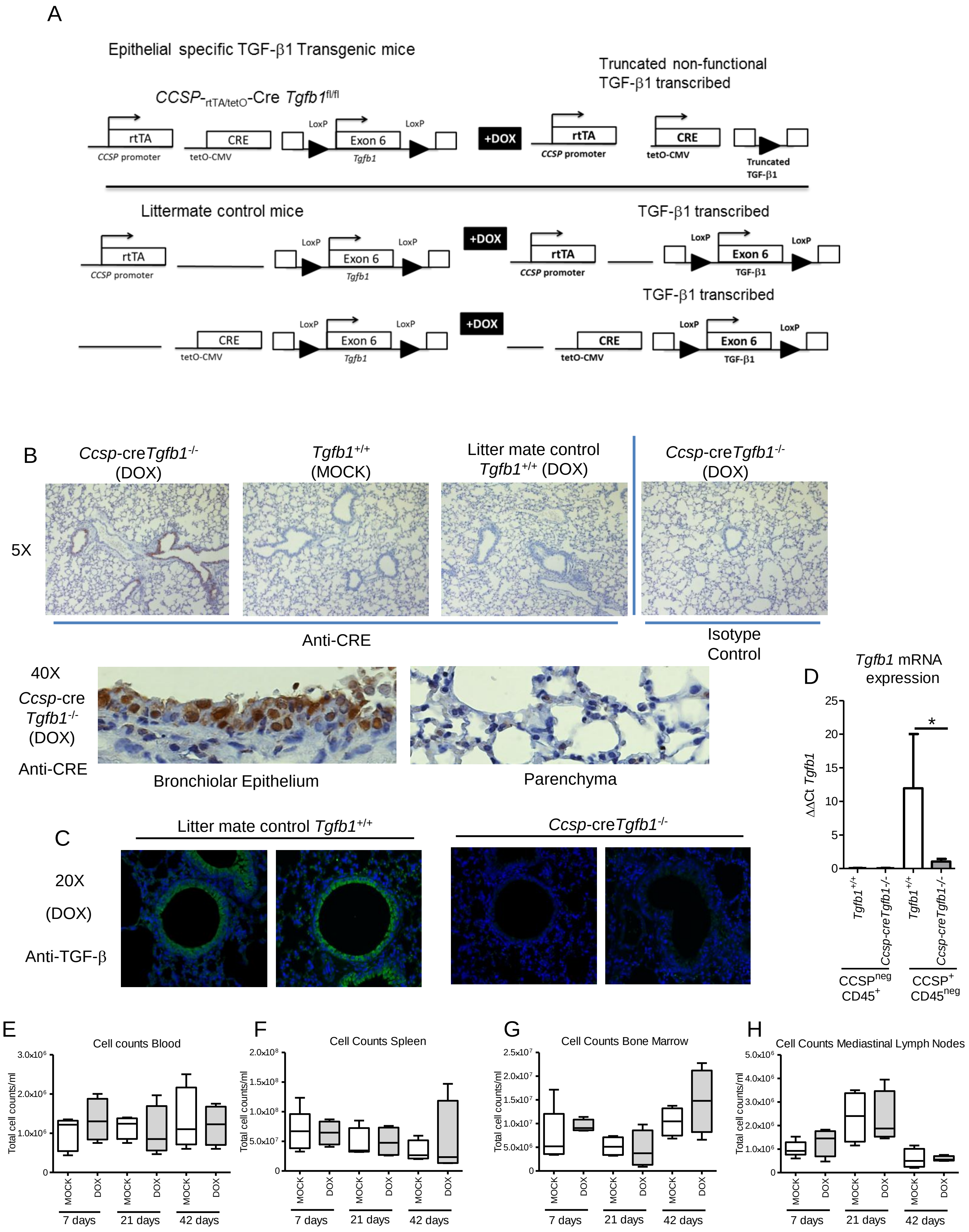
A
Epithelial specific TGF-β1 Transgenic mice
CCSP-rtTA/tetO-Cre Tgfb1fl/fl
Truncated non-functional TGF-β1 transcribed
rtTA
CCSP promoter
CRE
tetO-CMV
LoxP
Exon 6
Tgfb1
+DOX
Truncated TGF-β1
Littermate control mice
TGF-β1 transcribed
TGF-β1
B
Ccsp-creTgfb1-/- (DOX)
Tgfb1+/+ (MOCK)
Litter mate control Tgfb1+/+ (DOX)
5X
Anti-CRE
Isotype Control
40X
Ccsp-cre Tgfb1-/- (DOX)
Bronchiolar Epithelium
Parenchyma
C
Litter mate control Tgfb1+/+
Ccsp-creTgfb1-/-
20X
(DOX)
Anti-TGF-β
D
Tgfb1 mRNA expression
ΔΔCt Tgfb1
Tgfb1+/+
Ccsp-creTgfb1-/-
CCSPneg CD45+
CCSP+ CD45neg
E
Cell counts Blood
F
Cell Counts Spleen
G
Cell Counts Bone Marrow
H
Cell Counts Mediastinal Lymph Nodes
Total cell counts/ml
MOCK
DOX
7 days
21 days
42 days

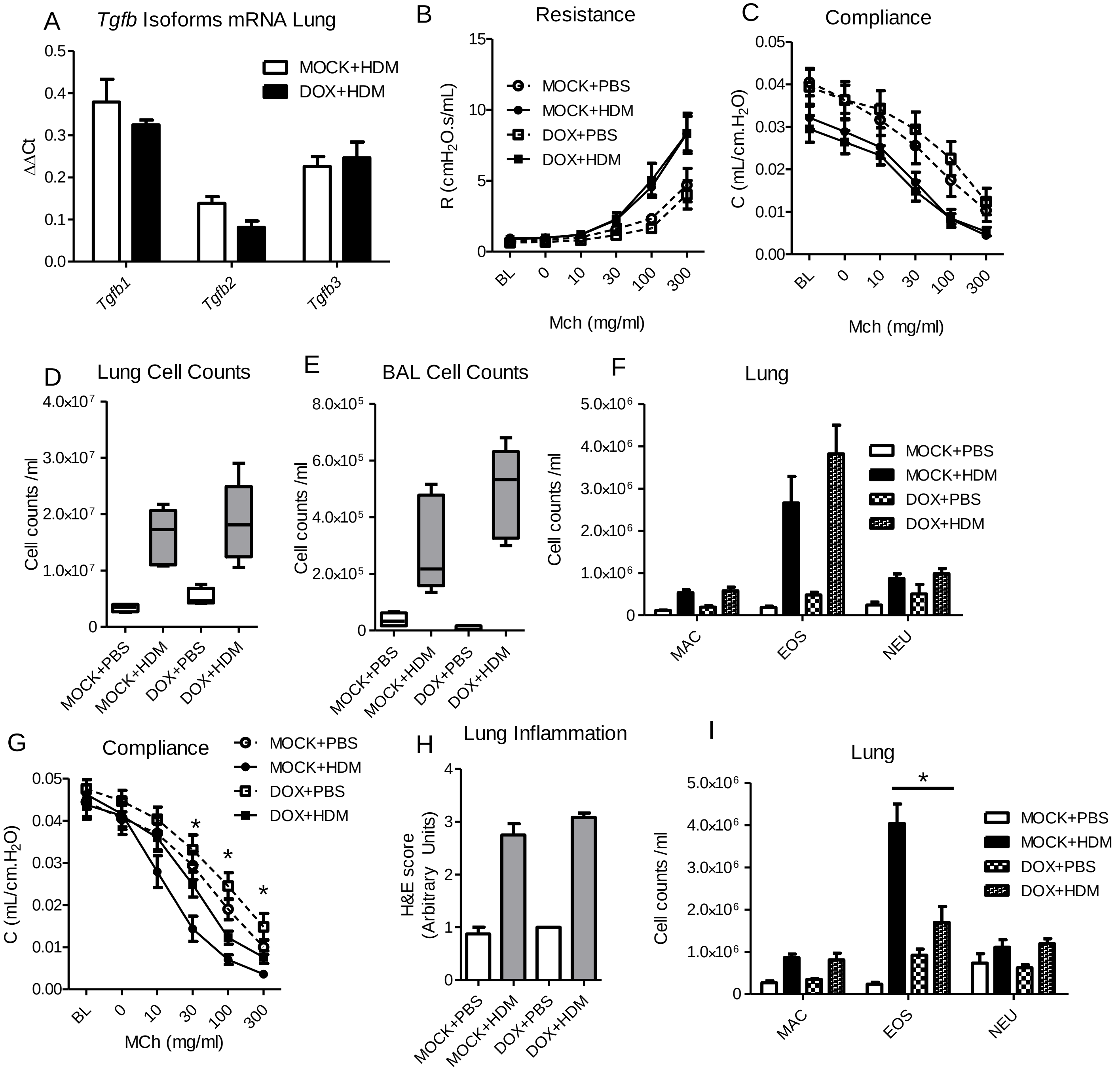

A
Tgfb Isoforms mRNA Lung
ΔΔCt
MOCK+HDM
DOX+HDM
Tgfb1
Tgfb2
Tgfb3
B
Resistance
R (cmH2O.s/mL)
MOCK+PBS
MOCK+HDM
DOX+PBS
DOX+HDM
Mch (mg/ml)
C
Compliance
C (mL/cm.H2O)
Mch (mg/ml)
D
Lung Cell Counts
Cell counts /ml
E
BAL Cell Counts
Cell counts /ml
F
Lung
Cell counts /ml
MAC
EOS
NEU
G
Compliance
C (mL/cm.H2O)
MCh (mg/ml)
H
Lung Inflammation
H&E score (Arbitrary Units)
I
Lung
Cell counts /ml

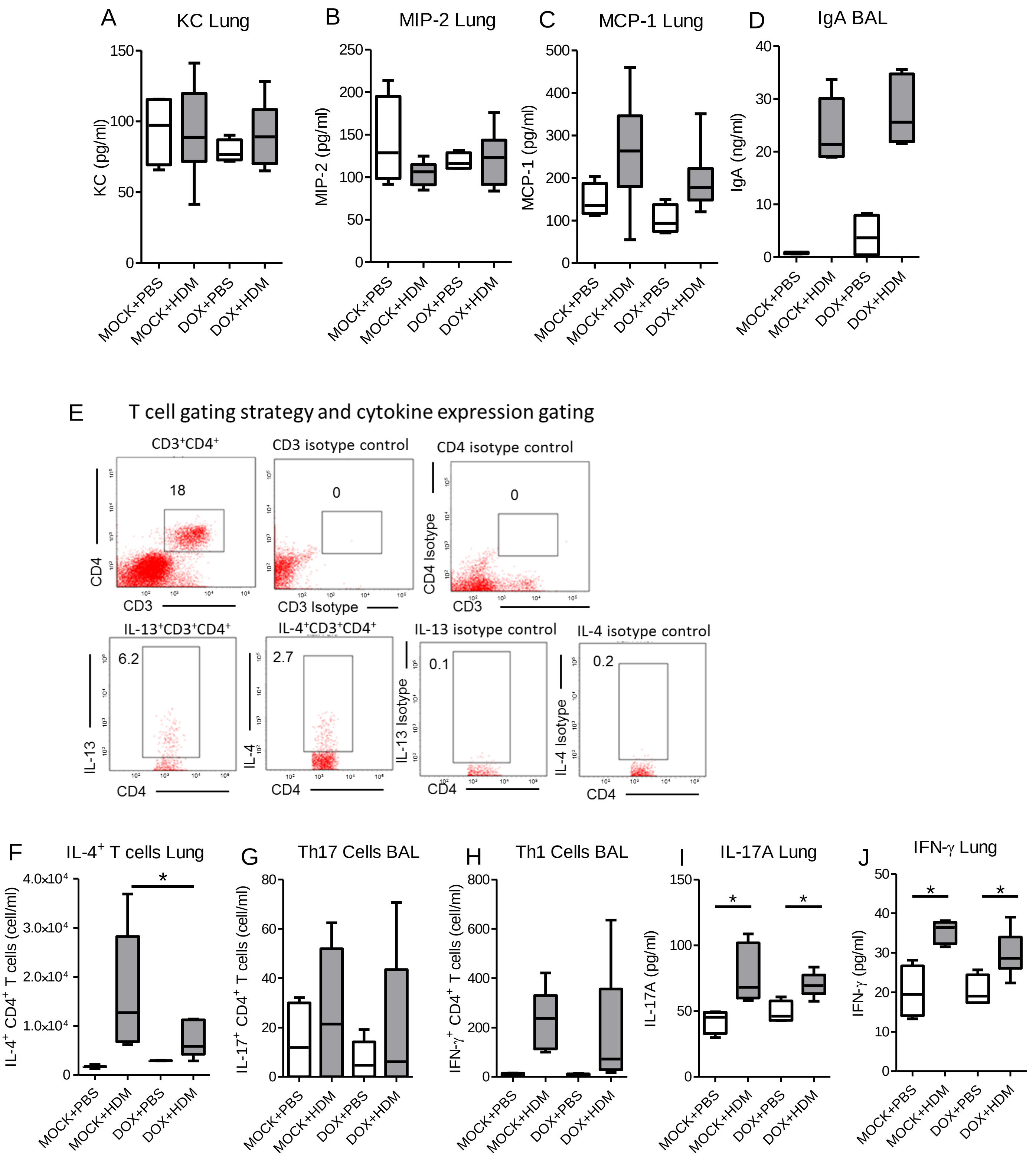

A
KC Lung
KC (pg/ml)
B
MIP-2 Lung
MIP-2 (pg/ml)
C
MCP-1 Lung
MCP-1 (pg/ml)
D
IgA BAL
IgA (ng/ml)
MOCK+PBS
MOCK+HDM
DOX+PBS
DOX+HDM
E
T cell gating strategy and cytokine expression gating
CD3+CD4+
18
CD3 isotype control
0
CD4 isotype control
0
CD4
CD3
CD3 Isotype
CD4 Isotype
IL-13+CD3+CD4+
6.2
IL-4+CD3+CD4+
2.7
IL-13 isotype control
0.1
IL-4 isotype control
0.2
IL-13
IL-4
IL-13 Isotype
IL-4 Isotype
F
IL-4+ T cells Lung
IL-4+ CD4+ T cells (cell/ml)
G
Th17 Cells BAL
IL-17+ CD4+ T cells (cell/ml)
H
Th1 Cells BAL
IFN-γ+ CD4+ T cells (cell/ml)
I
IL-17A Lung
IL-17A (pg/ml)
J
IFN-γ Lung
IFN-γ (pg/ml)
*

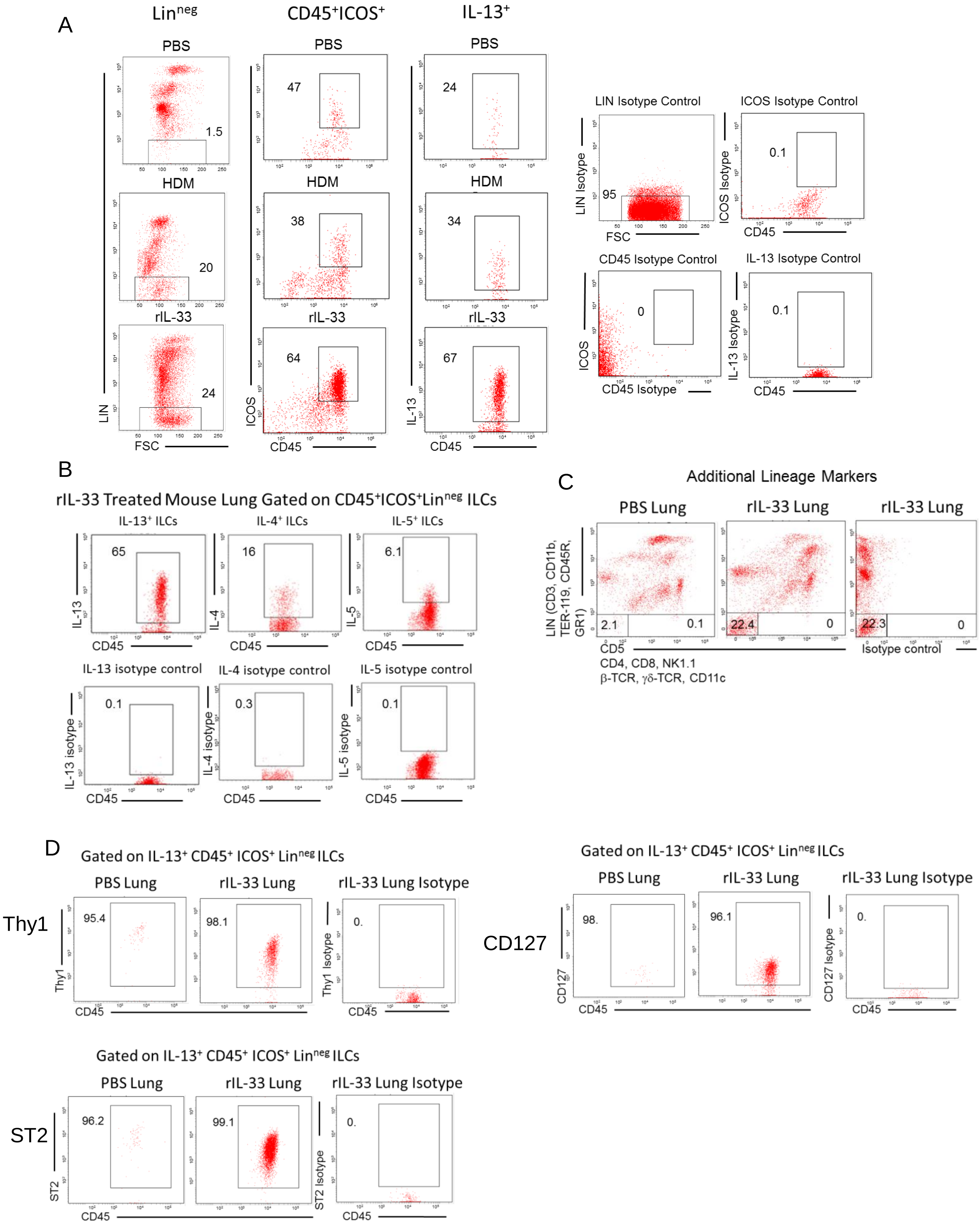
A
Lin^neg
CD45+ICOS+
IL-13+
PBS
HDM
rIL-33
1.5
20
24
47
38
64
24
34
67
LIN
FSC
ICOS
CD45
IL-13
LIN Isotype Control
ICOS Isotype Control
LIN Isotype
95
ICOS Isotype
0.1
CD45 Isotype Control
IL-13 Isotype Control
0
0.1
CD45 Isotype
IL-13 Isotype
B
rIL-33 Treated Mouse Lung Gated on CD45+ICOS+Lin^neg ILCs
IL-13+ ILCs
IL-4+ ILCs
IL-5+ ILCs
65
16
6.1
IL-4
IL-5
IL-13 isotype control
IL-4 isotype control
IL-5 isotype control
0.1
0.3
0.1
IL-13 isotype
IL-4 isotype
IL-5 isotype
C
Additional Lineage Markers
PBS Lung
rIL-33 Lung
rIL-33 Lung
LIN (CD3, CD11b, TER-119, CD45R, GR1)
2.1
0.1
22.4
0
22.3
0
CD5
CD4, CD8, NK1.1
β-TCR, γδ-TCR, CD11c
Isotype control
D
Gated on IL-13+ CD45+ ICOS+ Lin^neg ILCs
PBS Lung
rIL-33 Lung
rIL-33 Lung Isotype
Thy1
95.4
98.1
0.
Thy1 Isotype
CD127
98.
96.1
0.
CD127 Isotype
ST2
96.2
99.1
0.
ST2 Isotype

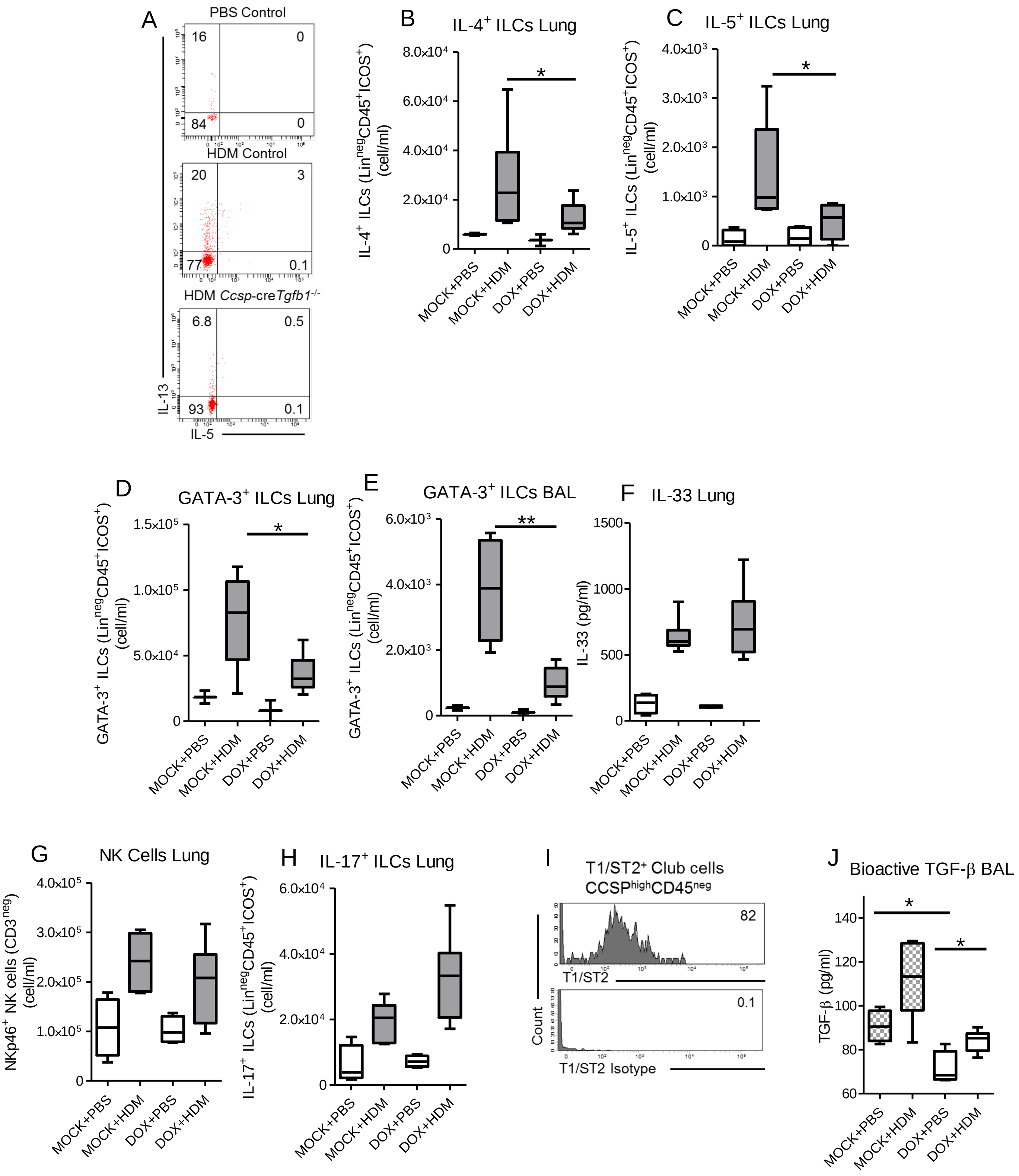
A
PBS Control
HDM Control
HDM Ccsp-cre Tgfb1-/-
IL-13
IL-5
B
IL-4+ ILCs Lung
IL-4+ ILCs (LinnegCD45+ICOS+) (cell/ml)
C
IL-5+ ILCs Lung
IL-5+ ILCs (LinnegCD45+ICOS+) (cell/ml)
D
GATA-3+ ILCs Lung
GATA-3+ ILCs (LinnegCD45+ICOS+) (cell/ml)
E
GATA-3+ ILCs BAL
F
IL-33 Lung
IL-33 (pg/ml)
G
NK Cells Lung
NKp46+ NK cells (CD3neg) (cell/ml)
H
IL-17+ ILCs Lung
IL-17+ ILCs (LinnegCD45+ICOS+) (cell/ml)
I
T1/ST2+ Club cells CCSPhighCD45neg
T1/ST2
T1/ST2 Isotype
Count
J
Bioactive TGF-β BAL
TGF-β (pg/ml)
MOCK+PBS
MOCK+HDM
DOX+PBS
DOX+HDM

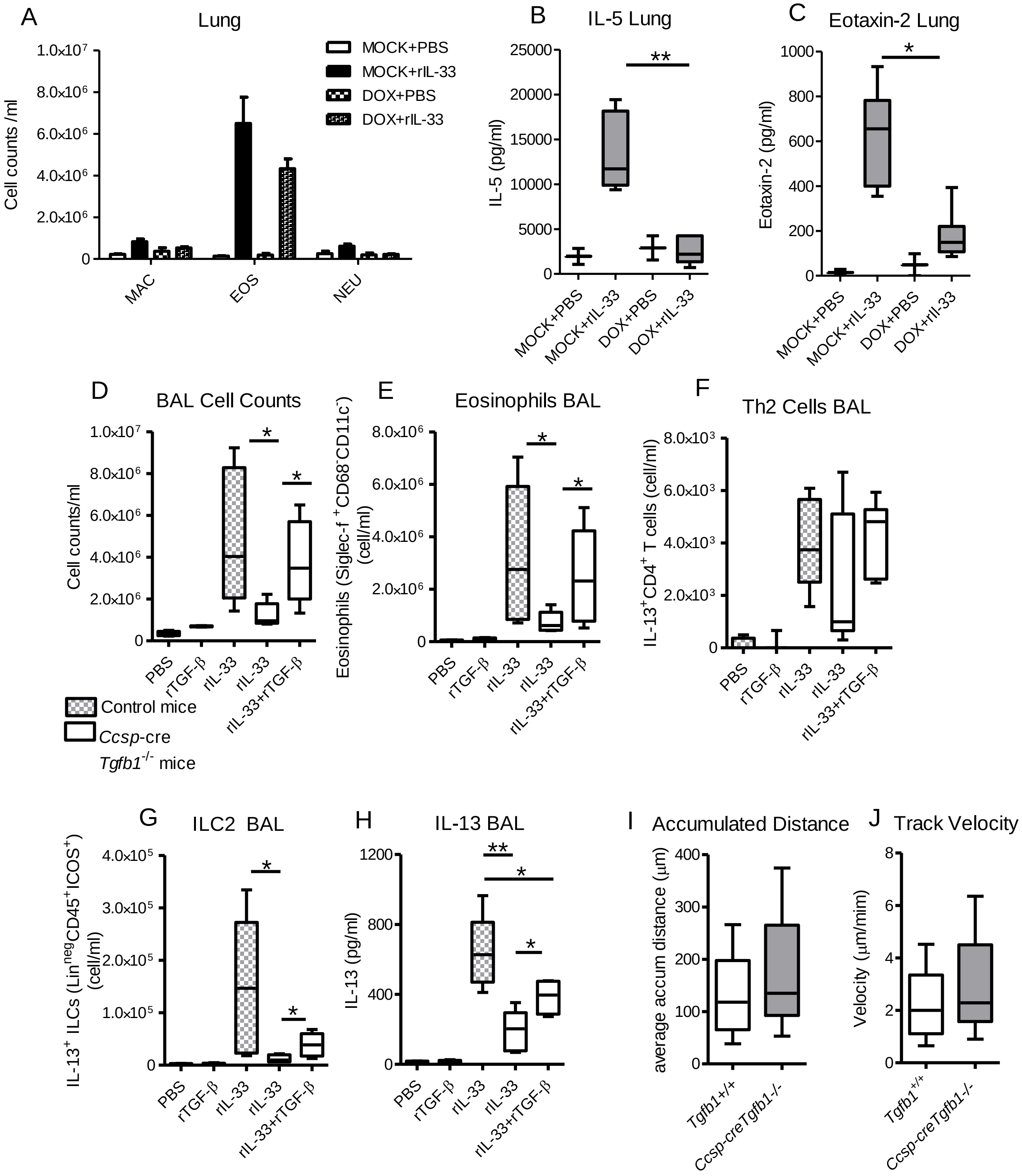

A
Lung
Cell counts /ml
MOCK+PBS
MOCK+rIL-33
DOX+PBS
DOX+rIL-33
MAC
EOS
NEU
B
IL-5 Lung
IL-5 (pg/ml)
MOCK+PBS
MOCK+rIL-33
DOX+PBS
DOX+rIL-33
C
Eotaxin-2 Lung
Eotaxin-2 (pg/ml)
MOCK+PBS
MOCK+rIL-33
DOX+PBS
DOX+rIl-33
D
BAL Cell Counts
Cell counts/ml
PBS
rTGF-β
rIL-33
rIL-33
rIL-33+rTGF-β
Control mice
Ccsp-cre Tgfb1-/- mice
E
Eosinophils BAL
Eosinophils (Siglec-f +CD68-CD11c-) (cell/ml)
F
Th2 Cells BAL
IL-13+CD4+ T cells (cell/ml)
G
ILC2 BAL
IL-13+ ILCs (LinnegCD45+ICOS+) (cell/ml)
H
IL-13 BAL
IL-13 (pg/ml)
I
Accumulated Distance
average accum distance (μm)
Tgfb1+/+
Ccsp-creTgfb1-/-
J
Track Velocity
Velocity (μm/mim)
Tgfb1+/+
Ccsp-creTgfb1-/-
